# *Clostridioides difficile* clinical diagnostic test methods and results are associated with recovery of *C. difficile* by stool culture

**DOI:** 10.1128/spectrum.03408-25

**Published:** 2026-01-09

**Authors:** Andrew M. Skinner, Alice Y. Guh, Laurica A. Petrella, Susan Sambol, Adam Cheknis, Stuart Johnson, Christopher A. Czaja, Helen Johnston, Elizabeth Basiliere, Robin A. Dhonau, Lauren Korhonen, Ashley L. Paulick, Matthew H. Samore, Michelle Adamczyk, Amy S. Gargis, Dale N. Gerding

**Affiliations:** 1 Research Section and Division of Infectious Diseases, VA Salt Lake City Health Care 20122 , Salt Lake City, Utah, USA; 2 Department of Medicine, Division of Infectious Diseases, School of Medicine, University of Utah, Salt Lake City, Utah, USA; 3 Centers for Disease Control and Prevention 1242, Atlanta, Georgia, USA; 4 Research Section, Edward Hines Jr., Veteran Affairs Hospital, Hines, Illinois, USA; 5 Colorado Department of Public Health and Environment, Denver, Colorado, USA; 6 Georgia Emerging Infections Program, Decatur, Georgia, USA; 7 Emory University School of Medicine 12239, Atlanta, Georgia, USA; 8 Division of Epidemiology, Department of Medicine, University of Utah School of Medicine 12348 , Salt Lake City, Utah, USA; 9 IDEAS Center of Innovation, VA Salt Lake City Healthcare System, Salt Lake City, Utah, USA; Tainan Hospital Ministry of Health and Welfare, Tainan, Taiwan

**Keywords:** epidemiology, *Clostridioides difficile*, clinical microbiology

## Abstract

Accurate molecular epidemiology relies on recovering *Clostridioides difficile* from clinical specimens and is essential for informing public health responses, characterizing strains that cause disease, and tracking strain prevalence. We performed a cross-sectional study of the recovery of *C. difficile* isolates from test-positive stools received January 2020 through December 2022 from the Colorado and Georgia Emerging Infections Program sites. Multivariable logistic regression models determined the adjusted odds ratio (aOR) and 95% confidence intervals (CIs) for factors which may influence *C. difficile* recovery in specimens that tested positive by either a dedicated or multiplex polymerase chain reaction (PCR) as part of either a reverse testing algorithm (PCR-positive, arbitrated by toxin enzyme immunoassay [EIA]) or a PCR-only testing protocol. Whole-genome sequencing was performed to determine the multilocus sequence types. *C. difficile* was recovered by culture from 84.3% (1,527/1,811) of all specimens. In specimens tested with a reverse testing algorithm, *C. difficile* culture recovery was less likely with multiplex versus dedicated PCR (aOR: 0.53; 95% CI: 0.34–0.85) and more likely in toxin EIA-positive versus toxin EIA-negative specimens (aOR: 8.07; 95% CI: 4.10–18.31). Recovery of *C. difficile* from stool cultures in PCR-only protocol cases was less likely when *C. difficile* was detected by multiplex PCR (aOR: 0.16; 95% CI: 0.03–0.55). ST1 was associated with toxin EIA-positive specimens, whereas ST2/110 was associated with toxin EIA-negative specimens. These findings can be used to maximize *C. difficile* surveillance programs, thereby enhancing the ability to generate robust molecular epidemiology data to inform prevention and control efforts.

**IMPORTANCE:**

Public health surveillance of *Clostridioides difficile* is essential for tracking strains, understanding transmission, and informing public health strategies. The foundation of the surveillance is the successful recovery of *C. difficile* from clinical specimens for molecular typing. This study reveals that the choice of diagnostic test significantly impacts the ability to recover *C. difficile* by culture. These data reveal that culture recovery was lower from specimens that tested positive by syndromic multiplex polymerase chain reaction (PCR) panels compared to dedicated *C. difficile* PCR assays. Furthermore, recovery was more than eight times more likely from toxin enzyme immunoassay (EIA)-positive specimens than from toxin EIA-negative specimens, with high-virulence strains, such as PCR-ribotype 027, being associated with toxin EIA-positive results. These findings demonstrate that common diagnostic practices could introduce biases into surveillance data, potentially misrepresenting the true prevalence and epidemiology of clinically important strains. Understanding these factors is crucial for optimizing surveillance programs to generate accurate molecular epidemiologic data.

## INTRODUCTION

*Clostridioides difficile* infection (CDI) surveillance, such as that conducted by the CDC’s Emerging Infections Program (EIP) since 2009, is essential to inform public health responses and characterize strains causing disease (1). The EIP cultures and types *C. difficile* from a convenience sample of *C. difficile*-positive stool specimens. These data inform national tracking of strain prevalence, clinical epidemiology, and national impact, making culture recovery critical to EIP surveillance (1–3).

The US Food and Drug Administration has approved numerous *C. difficile* clinical assays, including dedicated *C. difficile* polymerase chain reaction (PCR) assays and multiplex PCR platforms for the detection of multiple enteric pathogens, including *C. difficile* (4, 5). Due to the high sensitivity of *C. difficile* PCR-based tests and inability to distinguish colonization from infection, PCR-based assays are commonly utilized in multistep testing algorithms that include *C. difficile* toxin assays (6, 7). The Infectious Diseases Society of America and the Society for Healthcare Epidemiology of America recommend using a multistep algorithm over a dedicated PCR assay alone unless there are pre-agreed institutional criteria for patient stool submission (8). However, there is minimal guidance on how testing strategies can best inform culture practices to improve *C. difficile* recovery. To assess the association between *C. difficile* diagnostic test method and *C. difficile* culture recovery, we examined *C. difficile* data from EIP sites in Colorado and Georgia.

## MATERIALS AND METHODS

This is a cross-sectional study of *C. difficile* culture recovery from *C. difficile-*positive stool specimens collected at the Colorado and Georgia EIP sites from 1 January 2020 through 31 December 2022.

### Specimen selection and variable definitions

**Fig 1:**
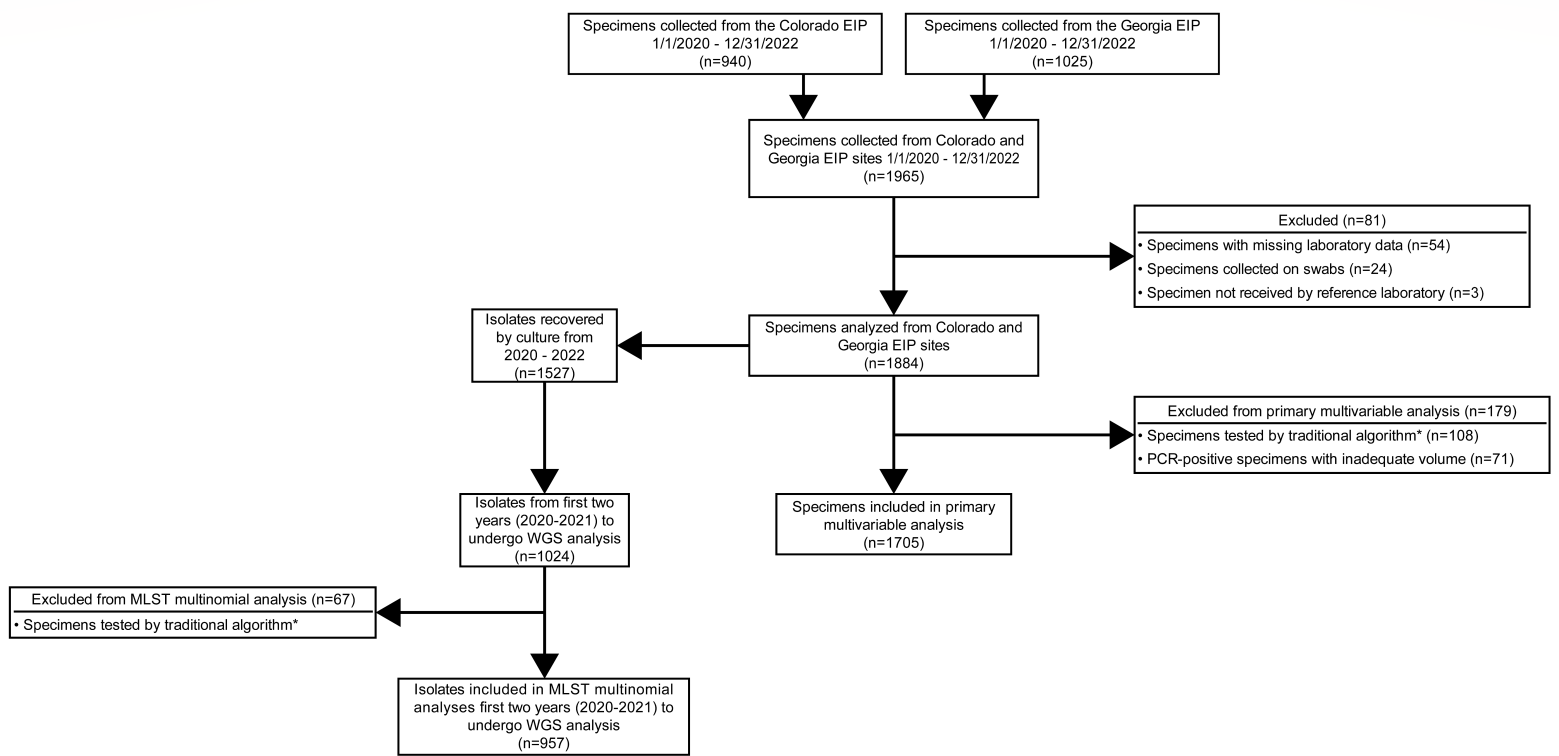
Flowsheet of isolate selection. WGS, whole-genome sequencing. The symbol “*” represents traditional algorithm defined as combined glutamate dehydrogenase (GDH) and toxin EIA.

*C. difficile* test-positive stool specimens were collected from clinical laboratories in Colorado, serving the Denver metropolitan area, and from clinical laboratories in Georgia, serving the Atlanta metropolitan area. Stool specimens were collected as part of clinical care as determined by providers at each healthcare facility. Specimens were processed and handled according to individual site protocols at each clinical laboratory (Tables S1 and S2). Specimens were collected as fresh stool, stool placed into Cary-Blair transport medium (CBTM), or swabs; however, swab specimens were excluded to ensure adequate volume for culture recovery and ensure generalizability between CBTM and fresh stool specimens (Fig. 1). Specimens that tested positive for *C. difficile* were shipped to the central EIP laboratory within their respective state before being batched and shipped to the Microbiologic Reference Laboratory (MRL) at the Edward Hines Jr. VA hospital for *C. difficile* culture and isolation. For analysis, *C. difficile* test-positive stool specimens were defined as specimens positive by any *C. difficile* assay regardless of whether the final test was negative for *C. difficile* (i.e., PCR-positive/toxin enzyme immunoassay [EIA]-negative).

Clinical tests to detect *C. difficile* were defined as a (i) *C. difficile*-targeted PCR assay or “dedicated PCR” (GeneXpert, Xpert *C. difficile*, Cepheid or BD Max Cdiff, Becton Dickerson Diagnostics), (ii) syndromic multiplex PCR panel or “multiplex PCR” (BioFire FilmArray Gastrointestinal Panel, bioMérieux), or (iii) GDH test paired with toxin EIA.

The PCR-only testing protocol was defined as a testing protocol in which stools were tested by either a dedicated or multiplex PCR, with no additional testing. Algorithmic testing protocols were defined as either a traditional testing algorithm or a reverse testing algorithm. The traditional algorithm was defined as stools tested by a GDH/toxin EIA, with discordant results (i.e., GDH-positive/toxin EIA-negative) arbitrated by a PCR. The reverse testing algorithm was defined as dedicated PCR-positive or multiplex PCR-positive specimens, followed by a subsequent toxin EIA assay, and were classified as either PCR-positive/toxin EIA-positive (PCR+/EIA+) or PCR-positive/toxin EIA-negative (PCR+/EIA−).

### Stool culture

Frozen stool specimens received at MRL were thawed, inoculated on taurocholate-cycloserine-cefoxitin-fructose agar (TCCFA) plates, and incubated at 36°C for 48–72 h in an anaerobic chamber (9). Specimens in which *C. difficile* was not recovered by culture and for which there was adequate remaining volume (>5 mL) were subjected to alcohol shock to enhance *C. difficile* recovery by plating to blood agar (10). Alcohol shock could not be performed on 73 specimens that lacked adequate volume. Purified cultures were placed into a glycerol freezing solution and frozen at −80°C.

### WGS

Because they lack a one-to-one relationship, multilocus sequence types (MLSTs) were grouped by associated PCR-ribotypes for statistical analysis (Table S3) (11). WGS methodology is described in the supplemental materials.

### Study outcomes

Descriptive statistics were provided for the laboratory characteristics of *C. difficile* test-positive stool specimens. The primary outcome of the study was *C. difficile* recovery by culture from *C. difficile* positive specimens tested by PCR-only or reverse testing algorithm protocols with adequate volume to undergo alcohol shock, if necessary. Secondary outcomes included the recovery of *C. difficile* on TCCFA plates, the requirement for alcohol shock (*n* = 443), and the determination of factors associated with key MLST groups.

### Statistical analysis

The *χ*2 test was used to assess differences in proportions across categorical variables where appropriate. Normality of continuous variables was determined by the Kolmogorov-Smirnov method. Non-parametric variables were reported as median with interquartile range (IQR) and were compared using the Wilcoxon signed-rank test.

A series of multivariable logistic regression models were constructed to determine the adjusted odds ratio (aOR) and 95% confidence intervals (CIs) for factors that may influence the recovery of *C. difficile* by culture in specimens that were dedicated PCR-positive or multiplex PCR-positive for *C. difficile*. Variables were selected *a priori* based upon the authors’ expertise and included the EIP state (Georgia or Colorado), collection year, testing algorithm, clinical test, toxin EIA status, individual laboratory ID, and the MRL technician. The individual laboratory ID was excluded due to high collinearity with the clinical test method (Fig. S2).

Multinomial logistic regression models were constructed to assess the association between key MLST groups and clinical testing characteristics. Model construction details are provided in the supplemental materials.

This activity was reviewed by CDC, deemed not research, and was conducted consistent with applicable federal law and CDC policy^§^ [§See e.g., 45 C.F.R. part 46, 21 C.F.R. part 56; 42 U.S.C. §241(d); 5 U.S.C. §552a; 44 U.S.C. §3501 et seq]. This activity was deemed either non-research or received Institutional Review Board (IRB) approval with a waiver of informed consent in participating EIP sites.

Statistical analysis was performed using RStudio version 4.3.2.

### *C. difficile* recovery in CBTM

Specimens tested by multiplex PCR are required to be submitted to the clinical laboratory in CBTM media, whereas specimens tested by dedicated PCR and GDH/toxin EIA have no such requirement. To validate the efficacy of CBTM for subsequent recovery of *C. difficile*, separate experiments were performed on anonymized frozen stool specimens (*n* = 20) in which *C. difficile* had previously been recovered as part of a non-EIP related study approved by the Edward Hines Jr., VA IRB (IRB no: 1583520). Culture methods and scoring are described in the supplemental materials (Fig. S1).

## RESULTS

A total of 1,965 *C*. *difficile* test-positive stool specimens were collected (940 Colorado; 1,025 Georgia). Specimens were excluded for missing clinical laboratory testing information (*n* = 54), were never received by the MRL (*n* = 3), or specimens were collected on a swab (*n* = 24), leaving 1,884 specimens for initial analysis (Fig. 1; see Tables S4 to S6 for excluded specimens).

### Basic laboratory data

Among the 1,884 *C. difficile* test-positive specimens, 49.7% (937) were from Colorado, and 50.3% (947) were from Georgia (Table 1). Among the 937 specimens collected from Colorado, the most common clinical diagnostic test was a multiplex PCR (72.6%, 680), and a PCR-only protocol was the most common testing protocol (74.4%, 697). Among the 947 specimens collected from Georgia, the most common clinical diagnostic test was a dedicated PCR (81.7%, 774), and a reverse testing algorithm was the most common protocol (82.4%, 780). Among the 1,020 specimens that underwent toxin EIA testing (i.e., traditional algorithm and reverse algorithm), 40.3% (411) were toxin EIA-positive. Among the 912 specimens that were *C. difficile* positive by a reverse testing algorithm protocol, 38.0% (273/719) of the dedicated PCR specimens, and 26.4% (51/193) of the multiplex PCR specimens were PCR+/toxin EIA+ (*P* < 0.01).

**TABLE 1:** Characteristics of *C. difficile* test-positive stool specimens from EIP surveillance in Colorado and Georgia, January 2020 through December 2022^*d*^

Variables	All specimens (*n* = 1,884)	Colorado (*n* = 937)	Georgia (*n* = 947)
Stool specimen collection year			
2020 (%)	565 (30.0%)	239/565 (42.3%)	326/565 (57.7%)
2021 (%)	689 (36.6%)	352/689 (51.1%)	337/689 (48.9%)
2022 (%)	630 (33.4%)	346/630 (54.9%)	284/630 (45.1%)
Stool specimen storage			
Frozen stool (%)	974 (51.7%)	238 (25.4%)	735 (77.8%)
Stool in CBTM (%)	910 (48.3%)	698 (74.6%)	210 (22.2%)
Initial clinical diagnostic test			
Dedicated PCR (%)	923 (49.0%)	149 (15.9%)	774 (81.7%)
Toxin EIA-positive (%)	273/719 (38.0%)	37/112 (33.0%)	236/607 (38.9%)
Multiplex PCR (%)	853 (45.3%)	680 (72.6%)	173 (18.3%)
Toxin EIA-positive (%)	51/193 (26.4%)	7/20 (35.0%)	44/172 (25.4%)
GDH (%)	108 (5.7%)	108 (11.5%)	0
Toxin EIA-positive (%)	87 (80.6%)	87 (80.6%)	0
Clinical testing strategy			
PCR-only testing strategy (%)	864 (45.9%)	697 (74.4%)	167 (17.6%)
Traditional testing algorithm^*a*^ (%)	108 (5.7%)	108 (11.5%)	0
Toxin EIA-positive (%)	87 (80.6%)	87 (80.6%)	0
Reverse testing algorithm^*b*^ (%)	912 (48.4%)	132 (14.1%)	780 (82.4%)
Toxin EIA-positive (%)	324 (35.6%)	44 (33.3%)	280 (35.9%)
Median time from collection to specimen receipt at reference laboratory (IQR) (days)	94 (45–142)	45 (36–56)	141 (124–176)
Collected from a patient with clinically documented diarrhea^*c*^	1,354/1,511 (89.6%)	722/787 (91.7%)	632/724 (87.3%)

^*a*^ Traditional testing algorithm: GDH/toxin EIA arbitrated by PCR.

^*b*^ Reverse testing algorithm: PCR followed by toxin EIA for PCR positives.

^*c*^ Diarrhea documented by local hospital.

^*d*^ CBTM, Cary-Blair transport medium; PCR, polymerase chain reaction; dedicated PCR, PCR specifically for *C. difficile*; multiplex PCR, PCR utilized for the detection of multiple gastrointestinal pathogens; GDH, glutamate dehydrogenase; EIA, enzyme immunoassay; IQR, interquartile range; TCCFA, taurocholate-cycloserine-cefoxitin-fructose agar.

### Recovery of *C. difficile* by culture

Among 1,884 specimens, *C. difficile* was recovered from 72.0% (1,357) on initial TCCFA culture. Of the 454 specimens not recovered by initial TCCFA, *C. difficile* was recovered from 37.4% (170) that underwent alcohol shock. After excluding the specimens with inadequate volume for complete testing (Fig. 1), 1,811 specimens remained for analysis, and *C. difficile* was recovered by culture from 84.3% (1,527) of specimens (Table 2).

**TABLE 2:** Recovery of *C. difficile* by culture from clinical stools that were positive for *C. difficile* identified through EIP surveillance in Colorado and Georgia, January 2020 through December 2022^*a*^

	All specimens	GDH	Dedicated PCR	Multiplex PCR	*P* value
Total recovery of *C. difficile*	1,527/1,884 (81.1%)	103/108 (95.4%)	804/923 (87.4%)	620/853 (72.7%)	<0.01
Recovery of *C. difficile* by TCCFA	1,357/1,884 (72.0%)	95/108 (88.0%)	738/923 (80.0%)	524/853 (61.4%)	<0.01
Recovery of *C. difficile* by alcohol shock	170/454 (37.5%)	8/11 (72.7%)	66/143 (46.2%)	96/300 (32.0%)	<0.01
Recovery of *C. difficile* in specimens with adequate volume	1,527/1,811 (84.3%)	103/106 (97.2%)	804/881 (91.4%)	620/824 (75.2%)	<0.01

^*a*^ CBTM, Cary-Blair transport medium; PCR, polymerase chain reaction; dedicated PCR, PCR specifically for *C. difficile*; multiplex PCR, PCR utilized for the detection of multiple gastrointestinal pathogens; GDH, glutamate dehydrogenase; EIA, enzyme immunoassay; IQR, interquartile range; TCCFA, taurocholate-cycloserine-cefoxitin-fructose agar.

Among specimens which had adequate volume for TCCFA and alcohol shock culture, *C. difficile* was recovered from 91.3% (804/881) of dedicated PCR-positive stool specimens compared to 75.2% (620/824) of specimens that were multiplex PCR-positive (*P* < 0.01).

### Multivariable logistic regression analysis of specimens positive by dedicated or multiplex PCR

In 1,705 specimens (Fig. 1), multiplex PCR-positive specimens had reduced odds of *C. difficile* recovery compared to dedicated PCR-positive specimens (aOR: 0.32; 95% CI: 0.22–0.46) (Table 3). Similar findings were noted for TCCFA culture recovery (aOR: 0.53; 95% CI: 0.40–0.70) (Table S7), and alcohol shock methods (aOR: 0.35; 95% CI: 0.19–0.62) (Table S8).

**TABLE 3:** Multivariable logistic regression model for *C. difficile* recovery from stool specimens positive by dedicated or multiplex PCR (*n* = 1,705)^*a*^

Variable	aOR (95% CI)
Initial clinical diagnostic test	
Dedicated PCR	REF
Multiplex PCR	0.32 (0.22–0.46)
EIP site	
Colorado	REF
Georgia	1.36 (0.87–2.10)
Clinical testing algorithm	
PCR-only testing protocol	REF
Reverse testing algorithm	0.82 (0.53–1.24)
Collection year	
2020	REF
2021	0.94 (0.66–1.35)
2022	1.11 (0.77–1.60)
MRL laboratory technician	
Laboratory technician #1	REF
Laboratory technician #2	1.03 (0.73–1.45)
Laboratory technician #3	0.82 (0.57–1.17)
Other laboratory technician	0.97 (0.45–2.35)

^*a*^ aOR, adjusted odds ratio; CI, confidence interval; REF, reference group; PCR, polymerase chain reaction; dedicated PCR, PCR specifically for *C. difficile*; multiplex PCR, syndromic PCR utilized for the detection of multiple gastrointestinal pathogens; EIA, enzyme immunoassay; EIP, Emerging Infections Program; MRL, Microbiological Reference Laboratory.

For specimens that were collected as part of a reverse testing algorithm, the odds of recovering *C. difficile* were reduced by 47% (aOR: 0.53; 95% CI: 0.34–0.85) for multiplex PCR-positive specimens when compared to dedicated PCR-positive specimens, and the odds of recovery were increased in toxin EIA-positive specimens (aOR: 8.07; 95% CI: 4.10–18.31) (Table 4). For specimens that were collected as part of a PCR-only testing protocol, the odds of *C. difficile* recovery were reduced by 84% for multiplex PCR-positive specimens (aOR: 0.16; 95% CI: 0.03–0.55) (Table 4).

**TABLE 4:** Multivariable logistic regression models for *C. difficile* recovery stratified by specimen collection algorithm for stool specimens positive by dedicated or multiplex PCR^*a*^

Reverse testing algorithm (*n* = 876)		PCR-only testing protocol (*n* = 829)	
	aOR (95% CI)^*a*^		aOR (95% CI)
Initial clinical diagnostic test		Initial clinical diagnostic test	
Dedicated PCR	REF	Dedicated PCR	REF
Multiplex PCR	0.53 (0.34–0.85)	Multiplex PCR	0.16 (0.03–0.55)
Toxin EIA status			
Toxin EIA-negative	REF		
Toxin EIA-positive	8.07 (4.10–18.31)		
EIP site		EIP site	
Colorado	REF	Colorado	REF
Georgia	0.82 (0.42–1.51)	Georgia	1.20 (0.17–5.23)
Year		Year	
2020	REF	2020	REF
2021	1.03 (0.55–1.95)	2021	0.90 (0.56–1.45)
2022	1.00 (0.58–1.75)	2022	1.14 (0.69–1.87)
MRL laboratory technician		MRL laboratory technician	
Laboratory technician #1	REF	Laboratory technician #1	REF
Laboratory technician #2	0.93 (0.54–1.63)	Laboratory technician #2	1.21 (0.78–1.87)
Laboratory technician #3	0.59 (0.30–1.13)	Laboratory technician #3	0.95 (0.60–1.50)
Other laboratory technician	0.80 (0.34–2.08)	Other laboratory technician	–^*b*^

^*a*^ aOR, adjusted odds ratio; CI, confidence interval; REF, reference group; PCR, polymerase chain reaction; dedicated PCR, PCR specifically for *C. difficile*; multiplex PCR, syndromic PCR utilized for the detection of multiple gastrointestinal pathogens; EIA, enzyme immunoassay; EIP, Emerging Infections Program; MRL, Microbiological Reference Laboratory.

^*b*^ "–” indicates that the PCR-only specimens were not handled by “Other Laboratory Technician”.

### MLST data

WGS was completed on 1,024 isolates from the first two study years. The most common MLST groups were ST42 (13.2%, 135), ST8 (9.8%, 100), ST2/110 (14.3%, 146), and ST1 (8.5%, 87) (Table S9).

Among the 67 isolates recovered from traditional algorithm specimens, regardless of toxin EIA results, the most common STs were: ST42 (10, 14.9%), ST1 (7, 10.4%), ST2/110 (6, 9.0%), and ST8 (5, 7.5%).

**Fig 2:**
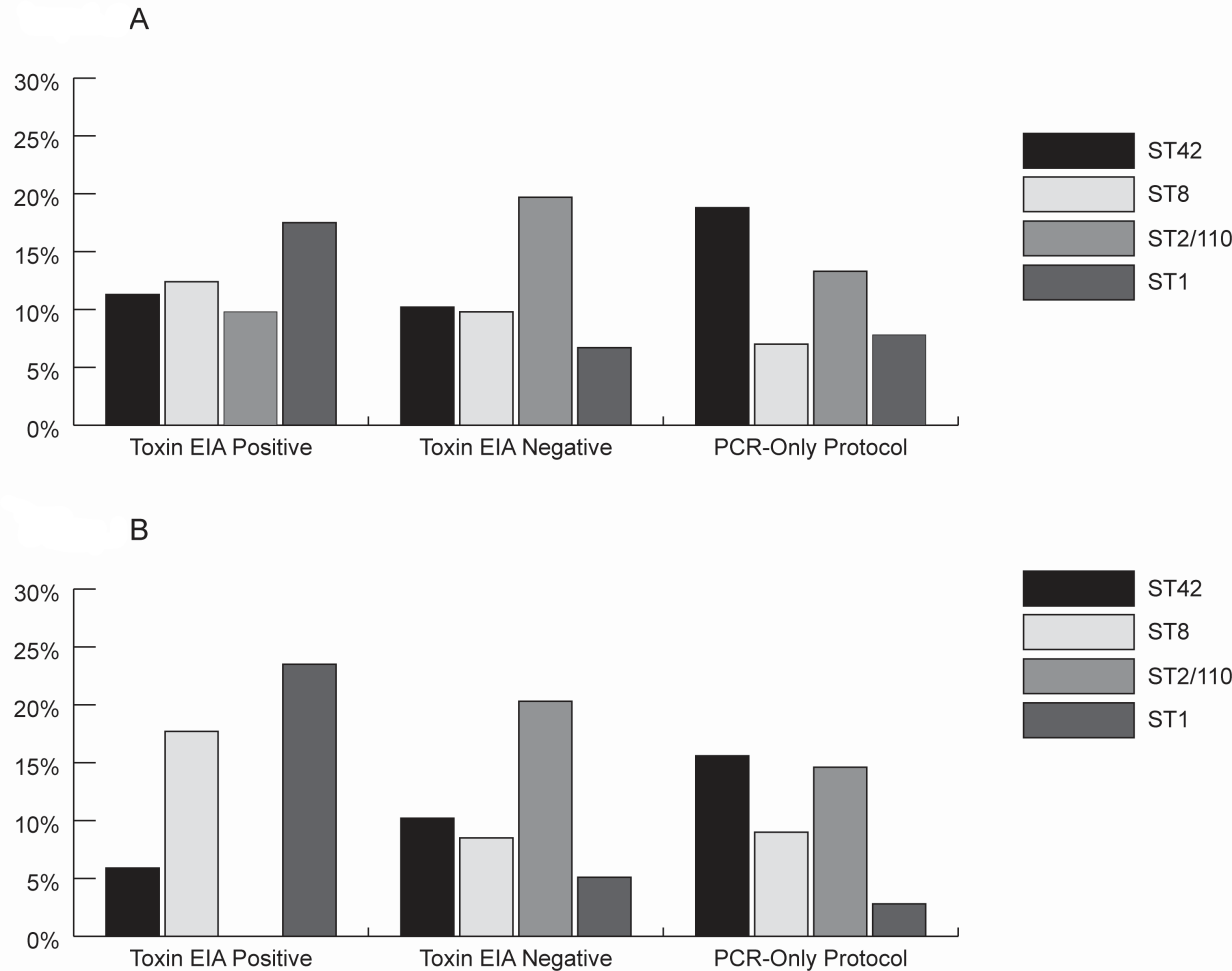
(**A**) *C. difficile* multilocus sequence typing of dedicated PCR specimens stratified by toxin EIA status. (**B**) *C. difficile* multilocus sequence typing of multiplex PCR specimens stratified by toxin EIA status. Prevalence of the four most common MLST groups, stratified by the initial PCR test and subsequent testing protocol. The *x*-axis on each chart displays three distinct specimen categories: (i) toxin EIA-positive (from reverse testing algorithm), (ii) toxin EIA-negative (from reverse testing algorithm), and (iii) PCR-only protocol.

Among the 128 specimens that underwent testing with a PCR-only protocol and were dedicated PCR-positive, the most common STs were ST42 (24, 18.8%), ST2/110 (17, 13.3%), ST1 (10, 7.8%), and ST8 (9, 7.0%) (Fig. 2A). Among 448 specimens that underwent testing with a reverse testing algorithm and were dedicated PCR-positive, the most common STs were ST2/110 (69, 15.4%), ST8 (49, 10.9%), ST42 (48, 10.7%), and ST1 (51, 11.4%).

After stratifying by toxin EIA status, ST1 accounted for 17.5% (34/194) of the PCR+/EIA+ specimens, whereas ST2/110 accounted for 9.8% (19/194). Conversely, ST2/110 accounted for 19.7% (50/254), and ST1 accounted for 6.7% (17/254) of the dedicated PCR+/EIA− specimens.

Among 288 specimens that underwent testing with a PCR-only protocol and were multiplex PCR-positive, the most common STs were: ST42 (45, 15.6%), ST2/110 (42, 14.6%), ST8 (26, 9.0%), and ST1 (8, 2.8%) (Fig. 2A). Among the 93 specimens that underwent testing with a reverse testing algorithm and were multiplex PCR-positive, the most common STs were: ST2/110 (12, 12.9%), ST1 (11, 11.8%), ST8 (11, 11.8%), and ST42 (8, 8.6%).

After stratifying by toxin EIA status, ST1 accounted for 23.5% (8/34) of the PCR+/EIA+ specimens, whereas there were no ST2/110 strains that were multiplex PCR+/EIA+ specimens. Conversely, ST2/110 accounted for 20.3% (12/59), and ST1 accounted for 5.1% (3/59) of the multiplex PCR+/EIA− specimens.

Multinomial models revealed that ST1 was 2.87 times (95% CI: 1.21–6.83) and 2.25 times (95% CI: 1.06–4.78) more likely to be recovered from specimens collected from Georgia over Colorado for ST42 and the “Other STs” groups, respectively (Table S10A). Additionally, ST1 was more likely to be recovered from toxin EIA-positive specimens compared to all other STs, and ST8 and ST42 were more likely to be recovered from toxin EIA-positive specimens compared to ST2/110. Conversely, these models revealed that ST2/110 isolates were more likely to be recovered from toxin EIA-negative specimens when compared to all other STs (Table S11A through D).

### Recovery of *C. difficile* from CBTM

Among the 20 stool *C. difficile* culture positive specimens that were placed into CBTM and PBS, the median recovery score was 3 (IQR: 1–3) (Fig. S1). The median recovery score for specimens that were placed into CBTM was 2.5 (IQR: 0.875–3.25), and the median recovery score for specimens that were placed into PBS was 3 (IQR: 0.875–3.0) (*P* = 0.88).

## DISCUSSION

Accurate molecular epidemiology has been essential in guiding the response to CDI for 30 years (12–17). However, as these data are reliant on recovery of *C. difficile* by culture, it is important to understand the factors that influence recovery. In our analysis, the odds of recovering *C. difficile* were higher in toxin EIA-positive specimens compared with toxin EIA-negative specimens and lower in specimens that were multiplex PCR-positive compared with dedicated PCR-positive specimens. These factors could have broad implications for *C. difficile* molecular and clinical epidemiology.

Previous data indicate that patients who are PCR+/EIA+ are more likely to suffer from recurrent CDI (18). Moreover, prior data link toxin EIA-positive status to higher toxin production strains (e.g., ST1) or higher bacterial burdens (19, 20). Our data support this finding, as the odds of recovering *C. difficile* from specimens that were toxin EIA-positive were eightfold higher than from specimens that were toxin EIA-negative.

Conversely, the odds of recovering *C. difficile* from toxin EIA-negative specimens are greatly reduced. While PCR+/EIA− results can indicate *C. difficile* colonization over CDI, recent data indicate lower 30-day mortality in treated versus untreated PCR+/EIA− patients, implying some have a true CDI (18, 21, 22). Thus, clinically significant toxin EIA-negative specimens exist. While delays in testing at the clinical laboratories could have resulted in toxin degradation and false-negative toxin EIA results, the significantly lower culture recovery in the toxin EIA-negative group supports the hypothesis that these specimens had lower organism burdens. Further studies are required to clarify if poor culture recovery from these specimens is due to a low bacterial burden or poor toxin EIA sensitivity, which could have significant implications for strain surveillance.

We found that *C. difficile* ST1 was more common in stool specimens with a toxin EIA-positive status, whereas ST2/110 was more common among toxin EIA-negative specimens. ST1 includes the high toxin-producing strain, RT027, implicated in multiple hospital outbreaks in North America and Europe, whereas ST2 and ST110 include the widely prevalent, but non-outbreak-associated RT014/020 strains. These data have infection control implications as ST1 groups are more frequently associated with antimicrobial resistance, whereas ST2 and ST110 isolates are commonly not associated with antimicrobial resistance (16, 23). This established link between MLST groups and antimicrobial resistance, when combined with our findings on toxin EIA status, presents additional surveillance opportunities. The established link between some MLST groups and antimicrobial resistance, combined with our data on toxin EIA status, suggests public health programs could use toxin EIA data as a potential proxy for monitoring the spread of clinically significant, resistant strains when culture-based surveillance is limited.

The increased sensitivity of certain clinical assays may further limit the ability to correctly interpret the molecular epidemiology of *C. difficile*. Initial studies of multiplex PCR platforms had suggested similar sensitivities and specificities compared to dedicated PCR assays, which previously demonstrated similar sensitivity and specificity to *C. difficile* toxigenic culture (24, 25). However, recent literature suggests that 19%–40% of stool specimens positive by multiplex PCR were GDH negative, whereas only 10% of stool specimens that were positive by dedicated PCR were GDH negative (26). This is concerning as GDH has been shown to have a negative predictive value of 97%–100% for recovery of *C. difficile* by toxigenic culture (26).

The MLST group distribution for specimens that were multiplex PCR and PCR-only protocol positive appears to be similar to specimens that were reverse testing algorithm positive, toxin EIA-negative, by favoring a lower prevalence of ST1 isolates and a higher prevalence of ST2/110. We hypothesize that lower culture recovery from multiplex PCR-positive specimens is multifactorial. While 23.5% of the multiplex-PCR specimens were found to have a viral or bacterial coinfection, we did not detect a significant impact on culture recovery. Therefore, we believe that multiplex PCR-tested specimens were from patients with a low pre-test probability for *C. difficile*, reflecting a lower organism burden and reducing *C. difficile* culture recovery, which surveillance programs may need to consider.

These data are in part supported by our assessment of the impact of CBTM on *C. difficile*, a requirement of the BioFire FilmArray Gastrointestinal assay (4). Previous metagenomic sequencing data revealed that stool specimens in CBTM that were left at ambient temperatures for up to 7 days did not negatively impact the firmicute population (27). Our semi-quantitative experiments further reinforce these findings, indicating that CBTM does not appear to have a deleterious effect on *C. difficile*.

A primary limitation of this study is the exclusion of contextual clinical data, such as antibiotic exposures or traditional *C. difficile* risk factors, collected by EIP surveillance. However, this approach was chosen to focus specifically on laboratory detection methods and reduce the risk of cognitive bias. Additionally, PCR cycle threshold (*C*_*T*_) data were not available for the analysis, which could reflect high or low bacterial burden. However, the primary multiplex PCR platform used in our study does not generate a quantitative *C*_*T*_ value and instead uses a melt curve for a qualitative result. The relatively poor recovery of *C. difficile* from multiplex PCR-positive specimens may reflect non-specific *C. difficile* testing (i.e., testing of patients lacking typical *C. difficile* risk factors), whereas the dedicated PCR was likely ordered when a provider had a strong suspicion for a CDI. However, testing preferences appear to reflect regional differences in laboratory practices, as multiplex PCR testing was more common in Colorado, whereas dedicated PCR was predominant in Georgia, though one laboratory in Georgia and two laboratories in Colorado used both (Tables S1 and S2). Additionally, recovery of *C. difficile* could have been improved by using an enriched broth, but it is unclear to what extent this would have improved, given the MRL’s use of an enriched agar (e.g., TCCFA) in conjunction with alcohol shock methods, but these methods may not be generalizable to less experienced laboratories (28). Notably, the study included three cryptic clade isolates which are typically PCR TcdB negative by conventional clinical assays (29). However, we cannot account for the possibility of mixed *C. difficile* populations, and future studies may require culture of multiple isolates from each specimen to account for the possibility of these mixed populations. Lastly, we could not control for variations in specimen handling across the clinical laboratories. However, as *C. difficile* recovery was stable through the study, pervasive handling issues were unlikely to have influenced our findings. Although the median time from collection to MRL receipt was longer in specimens collected from Georgia, recovery was also greater, indicating that time to culture does not impact recovery.

These results apply only to culture recovery of *C. difficile*; they do not suggest any clinical interpretation of test results for the treatment of patients. These findings suggest that incorporating toxin EIA testing into surveillance protocols may improve the understanding of circulating *C. difficile* strain epidemiology and better inform *C. difficile* prevention efforts, but further study is required.

## SUPPLEMENTAL MATERIAL

Supplemental Material spectrum.03408-25-s0001.docx Supplemental methods, Tables S1 to S11, and Fig. S1 and S2.

ASM does not own the copyrights to Supplemental Material that may be linked to, or accessed through, an article. The authors have granted ASM a non-exclusive, world-wide license to publish the Supplemental Material files. Please contact the corresponding author directly for reuse.
