# Supplementary material for: Clostridioides difficile clinical diagnostic test methods and results are associated with recovery of C. difficile by stool culture
Source: Microbiol Spectr. 2026 Jan 9;14(2):e03408-25. doi: 10.1128/spectrum.03408-25 (PMC12889085; doi:10.1128/spectrum.03408-25)
Supplement: Supplemental Material — Supplemental methods, Tables S1 to S11, and Fig. S1 and S2. [file spectrum.03408-25-s0001.docx]

Supplemental Documentation for *Clostridioides difficile* Clinical Diagnostic Test Methods and Results are Associated with Recovery of *C. difficile* by Stool Culture

[I. Description of specimen collection by laboratories 3](#_Toc202448666)

[**Supplemental Table 1** 3](#_Toc202448667)

[**Supplemental Table 2** 5](#_Toc202448668)

[II. Whole Genomic Sequencing Methods 7](#_Toc202448669)

[**Supplemental Table 3** 8](#_Toc202448670)

[III. Recovery of C. difficile in Cary-Blair Transport Medium Methods 9](#_Toc202448671)

[**Supplemental Figure 1** 9](#_Toc202448672)

[IV. Characteristics, recovery, and multilocus sequence testing (MLST) for specimens excluded from analysis 10](#_Toc202448673)

[**Supplemental Table 4** 10](#_Toc202448674)

[**Supplemental Table 5** 11](#_Toc202448675)

[**Supplemental Table 6** 11](#_Toc202448676)

[V. Multivariable Logistic Regression Model Variable Selection and Construction 12](#_Toc202448677)

[**Methods** 12](#_Toc202448678)

[**Supplemental Figure 2** 12](#_Toc202448679)

[**Logistic regression model for specimens positive by either dedicated PCR or multiplex PCR** 14](#_Toc202448680)

[**Logistic regression model for specimens positive by a reverse testing algorithm** 14](#_Toc202448681)

[**Logistic regression model for specimens positive by a PCR-only protocol** 14](#_Toc202448682)

[**Additional logistic regression results for secondary outcomes (TCCFA and alcohol shock recovery)** 14](#_Toc202448683)

[**Supplemental Table 7** 15](#_Toc202448684)

[**Supplemental Table 8** 15](#_Toc202448685)

[VI. Multiplex PCR Coinfection Data 16](#_Toc202448686)

[**Statistical Analysis** 16](#_Toc202448687)

[VII. MLST data 17](#_Toc202448688)

[**Supplemental Table 9** 17](#_Toc202448689)

[VIII. Multinomial Logistic Regression Analysis 20](#_Toc202448690)

[**Methods** 20](#_Toc202448691)

[**Multinomial Logistic Regression Models** 20](#_Toc202448692)

[**Results** 20](#_Toc202448693)

[**Supplemental Table 10A** 21](#_Toc202448694)

[**Supplemental Table 10B** 21](#_Toc202448695)

[**Supplemental Table 10C** 21](#_Toc202448696)

[**Supplemental Table 10D** 22](#_Toc202448697)

[**Supplemental Table 11A** 23](#_Toc202448698)

[**Supplemental Table 11B** 24](#_Toc202448699)

[**Supplemental Table 11C** 25](#_Toc202448700)

[**Supplemental Table 11D** 26](#_Toc202448701)

[IX. Supplemental Documentation References 27](#_Toc202448702)

**I. Description of specimen collection by laboratories**

**Supplemental Table 1**

Stool specimen testing and handling practices in seven Colorado clinical laboratories, EIP CDI surveillance, January 2020 - December 2022

|  | Lab #1 | Lab #2 | Lab #3 | Lab #4 | Lab #5 | Lab #6 | Lab #7 |
| --- | --- | --- | --- | --- | --- | --- | --- |
| Total number of stool specimens | 1 | 35 | 273 | 8 | 71 | 367 | 185 |
| Number of specimens excluded from analysis | 0 | 0 | 0 | 0 | 1 | 2 | 0 |
| Reason for exclusion | -- | -- | -- | -- | Specimen not received by reference laboratory (n=1) | Unclear clinical tests used for *C. difficile* detection (n=1)  Specimen not received by reference laboratory (n=1) | -- |
| Test algorithm | PCR-only algorithm | Reverse algo for inpatient tests (n=32)  PCR for outpatient or ileus (n=3) | Traditional algorithm inpatient tests (n=38)  PCR-only algorithm for outpatient (n=235) | Reverse algo for inpatient tests (n=4)  PCR for outpatient or ileus (n=4) | Traditional algorithm | PCR-only testing algorithm | Reverse algo for inpatient tests (n=96)  PCR for outpatient or ileus (n=89) |
| Clinical detection test | Dedicated PCR | Dedicated PCR | Traditional algorithm completed by GDH/Toxin EIA with discordant results discriminated by dedicated PCR  PCR-only algorithm completed by multiplex PCR | Dedicated PCR | Traditional algorithm completed by GDH/Toxin EIA with discordant results discriminated by dedicated PCR | Multiplex PCR (n=343)  Dedicated PCR (n=23)  Unknown (n=1) | Multiplex PCR (n=102)  Dedicated PCR (n=83) |
| How long are specimens held between collection and testing? | Unknown | Unknown | 0 – 24 hours | unknown | 0 – 36 hours* | 0 – 24 hours | 0 – 24 hours |
| In what conditions are specimens held between collection and testing? | Unknown | Unknown | Fresh stool is tested immediately. Refrigerated if arriving from satellite facility | Unknown | Fresh stool is tested immediately. Refrigerated if arriving from satellite facility | Refrigerated or frozen until testing completed. | Refrigerated until testing. |
| In what conditions are specimens held between testing and shipping to EIP lab? | Refrigerated at 2-8°C | Refrigerated at 2-8°C | Frozen at  -20°C | Refrigerated at 2-8°C | Refrigerated at 2-8°C | Refrigerated at 2-8°C | Refrigerated at 2-8°C |
| How often are specimens shipped to the EIP lab? | Monday - Friday | Monday – Friday | Monday - Friday | Unknown | Monday - Friday | Monday - Friday | Monday - Friday |
| Are specimens shipped daily or in batch to the EIP lab? | Daily | Daily | Daily | Unknown | Daily | Daily | Daily |
| In what conditions are the specimens shipped to the EIP lab? | On cold pack | Unknown | On cold pack | Unknown | On cold pack | On cold pack | On cold pack |
| How are specimens processed when they arrive at the EIP lab? | If the specimen arrives at state Lab before 4 p.m. it is frozen at -20°C within 4 hours. If it arrives after 4 p.m. it is refrigerated overnight and frozen the next day. | | | | | | |
| How long are specimens held between arrival at the EIP lab and shipping to the MRL? | Up to 4 months and sent in batch. | | | | | | |
| In what conditions are specimens held between arrival at the EIP lab and shipping to the MRL? | Specimens are maintained in -20°C freezer | | | | | | |
| * Lab processed stool specimens Monday – Saturday. If the specimen arrived at lab after 4PM on Saturday, was not processed until following Monday morning.  MRL: Microbiologic Reference Laboratory | | | | | | | |

**Supplemental Table 2**

Stool specimen testing and handling practices in eight Georgia clinical laboratories, EIP CDI surveillance, January 2020 - December 2022

|  | Lab #1 | Lab #2 | Lab #3 | Lab #4 | Lab #5 | Lab #6 | Lab #7 | Lab #8 | |
| --- | --- | --- | --- | --- | --- | --- | --- | --- | --- |
| Total number of stool specimens | 77 | 440 | 92 | 36 | 215 | 163 | 1 | 1 | |
| Number of specimens excluded from analysis | 15 | 1 | 20 | 36 | 5 | 0 | 0 | 1 | |
| Reason for exclusion | Specimen received on swab (n=15) | Specimen not received by reference lab (n=1) | Specimen received on swab (n=5)  Unclear clinical tests used for *C. difficile* detection (n=15) | Unable to confirm toxin EIA results (n= 36) | Specimen received on swab (n=4)  Unable to confirm toxin EIA result (n =1) | -- | -- | Unclear clinical tests used for *C. difficile* detection (n=1) | |
| Test algorithm | Reverse testing algorithm | Reverse testing algorithm | Reverse testing algorithm | Traditional algorithm | Reverse testing algorithm | PCR-only testing algorithm | Reverse testing algorithm | Reverse testing algorithm | |
| Clinical detection test | Dedicated PCR | Dedicated PCR | Dedicated PCR | GDH/Toxin EIA with discordant results discriminated by dedicated PCR | Dedicated PCR (n=41) and Multiplex PCR (n=174) | Dedicated PCR | Dedicated PCR | Unknown | |
| How long are specimens held between collection and testing? | 0 – 24 hours | 0 – 24 hours | 0 – 24 hours | 0 – 24 hours | 0 – 24 hours | 0 – 36 hours* | Unknown | 0 – 1 hour | |
| In what conditions are specimens held between collection and testing? | Refrigerated until testing completed. | Refrigerated until testing completed. | Refrigerated until testing completed. | Fresh stool is tested immediately. | Fresh stool is tested immediately. | Refrigerated until testing completed. | Unknown | Either refrigerated or frozen | |
| In what conditions are specimens held between testing and shipping to EIP lab? | Frozen at  -20°C | Refrigerated at 2-8°C | Frozen at  -20°C | Frozen at  -20°C | Refrigerated at 2-8°C | Frozen at  -20°C | Refrigerated at 2-8°C | Refrigerated at 2-8°C | |
| How often are specimens shipped to the EIP lab? | Monthly | Twice weekly | Every 3-4 weeks | Every 2-3 months | Twice weekly | Every 2-3 months | Twice weekly | Twice weekly | |
| Are specimens shipped daily or in batch to the EIP lab? | Batch | Batch | Batch | Batch | Batch | Batch | Batch | Batch | |
| In what conditions are the specimens shipped to the EIP lab? | On dry ice | On cold pack | On dry ice | On dry ice | On cold pack | On dry ice | On cold pack | On cold pack | |
| How are specimens processed when they arrive at the EIP lab? | Specimens are placed into -20°C freezer on arrival | | | | | | | | |
| How long are specimens held between arrival at the EIP lab and shipping to the MRL? | Up to 6 months and sent in batch. | | | | | | | | |
| In what conditions are specimens held between arrival at the EIP lab and shipping to the MRL? | Specimens are maintained in -20°C freezer | | | | | | | | |
| * Lab processed stool specimens Monday – Saturday. If the specimen arrived at lab after 4PM on Saturday, was not processed until following Monday morning.  MRL: Microbiologic Reference Laboratory | | | | | | | | |  |

**II. Whole Genomic Sequencing Methods**

Whole genome sequencing (WGS) and analysis was performed as previously described.[1] Briefly, genomic DNA was extracted from colonies cultured overnight on CDC anaerobic blood agar with vitamin K, hemin, and 5% sheep blood under anaerobic conditions at 37°C. DNA was isolated using the Promega Maxwell 16-cell low elution volume DNA purification kit and the Maxwell 16 MDx instrument (Madison, WI). For WGS using the MiSeq system (Illumina, San Diego, CA), DNA was sheared using the Covaris ME220 focused ultrasonicator (Woburn, MA), and indexed libraries were prepared using the NuGEN Ovation ultralow system version 2 assay kit (San Carlos, CA) and the PerkinElmer Zephyr G3 next-generation sequencing (NGS) workstation (Waltham, MA). Libraries were analyzed using the standard-sensitivity NGS fragment analysis kit and fragment analyzer system (Agilent Technologies, Santa Clara, CA). Sequencing was performed using the MiSeq reagent kit version 2 (500 cycles) and MiSeq system (Illumina, San Diego, CA), generating 2 × 250-bp paired-end reads. Removal of adaptors from raw sequences was done using Trimmomatic version 0.36, and de novo assembly using SPAdes version 3.13.0.[2,3] For WGS using the NovaSeq system (Illumina, San Diego, CA), libraries were prepared from genomic DNA using Illumina DNA prep reagents (Illumina, San Diego, CA) and barcoding indices synthesized in the CDC Biotechnology Core Facility. The libraries underwent QC, normalization and pooling, and the final pool was sequenced using Illumina Novaseq 6000 SP Reagent kit v1.5 (500 cycles) (Illumina, San Diego, CA). Sequencing reads were filtered for read quality, basecalled and demultiplexed using bcl2fastq (v2.20). Sequence types were assigned using the software package MLST version 2.16 and the pubMLST *C. difficile* MLST scheme[4,5].

**MLST grouping for analysis**

Historically, ST1 commonly aligns with RT027, ST8 with RT002, and ST42 with RT106.[6–9] However, multiple RTs make up ST2 and ST110.[6] In 2018, the three RT groups which accounted for the majority of Emerging Infections Program (EIP) ST2 isolates were RT076 (41/127 [32.3%]), RT020 (35/127 [27.6%]), and RT014 (21/127 [16.5%]). In 2018, RT020, RT014, and RT076 accounted for 19/37 (51.4%), 3/37 (8.1%), and 3/37 (8.1%) of EIP isolates identified as ST110, respectively. Analysis of the available EIP ribotyping data from 2012 – 2018 revealed that from 2012 – 2016, RT014 and RT020 accounted for 5.8% (95% CI: 5.3% - 6.4%) and 6.1% (95% CI: 5.5% - 6.7%) of *C. difficile* cases documented by the EIP, respectively.[10] From 2017 – 2018, RT076 prevalence increased. RT014, RT020, and RT076 accounted for 5.9% (95% CI: 5.0% - 6.9%), 5.0% (95% CI: 4.1% - 5.9%), and 4.3% (95% CI: 3.4% - 5.1%) of *C. difficile* cases documented by the EIP, respectively.[10] Given the significant cross-over between RTs within ST2 and ST110, ST2 and ST110 were combined into a single variable to represent RT014, RT020, and RT076. (Supplemental Table 3)

**Supplemental Table 3**

*Clostridioides difficile* multilocus sequence types (MLST) and associated PCR-Ribotype groups used in this study.[6]

| Sequence type (ST) | PCR-Ribotype group |
| --- | --- |
| ST1 | RT027 |
| ST2/110 | RT014, RT020, RT076 |
| ST8 | RT002 |
| ST42 | RT106 |
| Other STs* | All other RTs |
| * Consists of 95 other ST groups which account for 54.2% of all isolates | |

**III. Recovery of C. difficile in Cary-Blair Transport Medium Methods**

Stool specimens were placed into CBTM and phosphate-buffered saline (PBS) separately at a dilution of 1:10. Solutions were left at ambient room temperature (18°C to 24°C) for either 4 hours or 24 hours. Specimens were subsequently refrigerated at 4°C for 7 days and then frozen at -20°C for an additional 5 days. Frozen solutions were thawed and 50 µl was inoculated onto TCCFA plates. *C. difficile* was streaked for isolation into four quadrants using a 10µl inoculation loop (Becton Dickson, Franklin Lakes, NJ). TCCFA plates were incubated anaerobically at 36°C for 48 hours. Plates were evaluated after incubation and a score was assigned to each isolate based on *C. difficile* growth per each quadrant and recovery in CBTM compared to PBS. (Supplemental Figure 1).

**
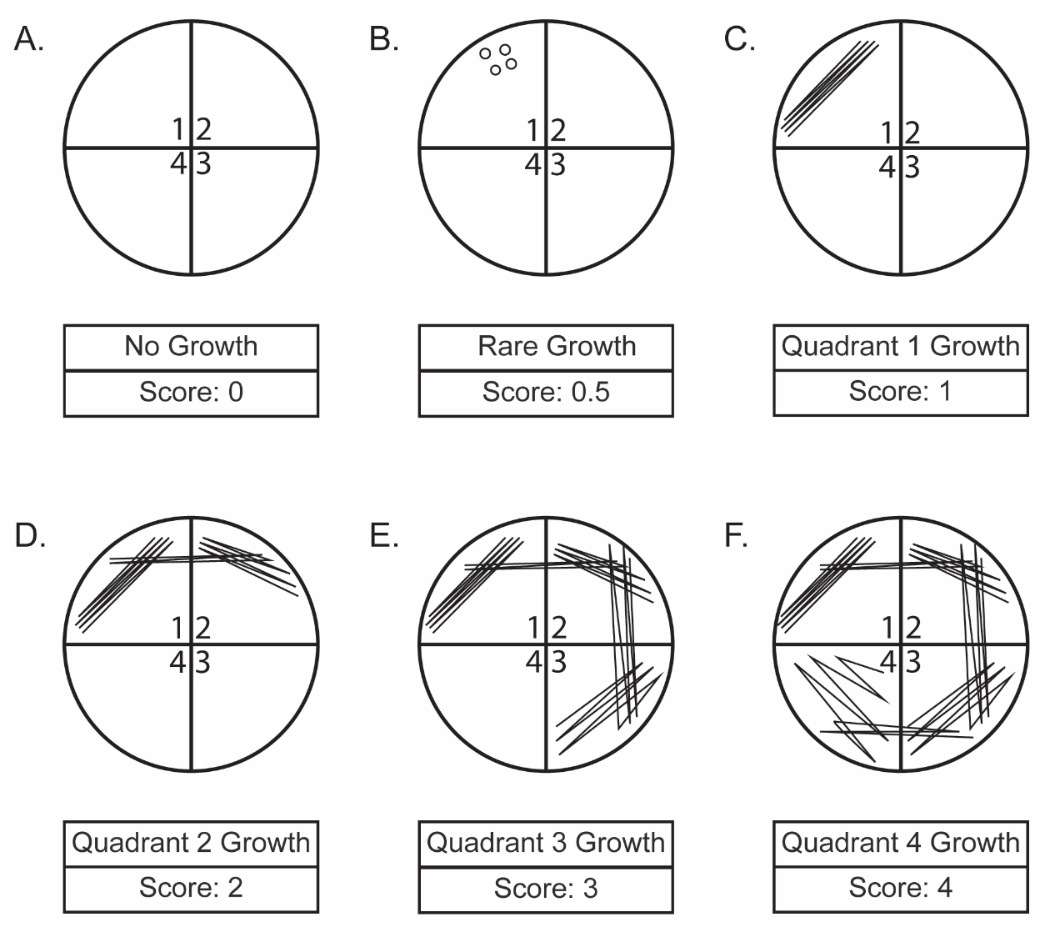
Supplemental Figure 1**

**Supplemental Figure 1 Legend**

*Panel A*: No Growth. **Score = 0;**

*Panel B*: Rare colonies noted but no confluent growth. **Score = 0.5**

*Panel C*: Confluent growth in quadrant 1. **Score = 1;**

*Panel D*: Confluent growth in quadrants 1 and 2. **Score = 2**

*Panel E*: Confluent growth in quadrants 1,2 and 3. **Score = 3**

*Panel F*: Confluent growth in quadrants 1,2,3, and individual colonies in quadrant 4. **Score = 4**

**IV. Characteristics, recovery, and multilocus sequence testing (MLST) for specimens excluded from analysis**

**Supplemental Table 4**

Characteristics of stool specimens from *Clostridioides difficile* test-positive stools identified through Emerging Infections Program surveillance in Colorado and Georgia, January 2020 through December 2022 excluded from the study.

|  | All Specimens (n=81) | Colorado (n=3) | Georgia (n=78) |
| --- | --- | --- | --- |
| **Variables** |  |  |  |
| Stool specimen collection Year |  |  |  |
| 2020 – k/n (%) | 30 (37.0%) | 1/30 (3.3%) | 29/30 (96.7%) |
| 2021– k/n (%) | 20 (24.7%) | 0/20 (0%) | 20/20 (100%) |
| 2022– k/n (%) | 31 (38.3%) | 2/31 (6.5%) | 29/31 (93.5%) |
| Stool specimen storage |  |  |  |
| Frozen Stool– k/n (%) | 53 (65.4%) | 1 (33.3%) | 52 (66.7%) |
| Stool in CBTM– k/n (%) | 3 (8.9%) | 1 (33.3%) | 2 (2.6%) |
| Swab specimen – k/n (%) | 24 (29.6%) | 0 (0%) | 24 (30.8%) |
| Unknown – k/n (%) | 1 (1.2%) | 1 (33.3%) | 0 (0%) |
| Initial clinical diagnostic test |  |  |  |
| Dedicated PCR– k/n (%) | 25 (30.9%) | -- | 25 (32.1%) |
| Toxin EIA positive (%) | 13/25 (52.0%) | -- | 13/25 (52.0%) |
| No toxin EIA testing done (%) | 0/25 (0%) | -- | 0/25 (0%) |
| Multiplex PCR– k/n (%) | 3 (3.7%) | 1 (33.3%) | 2 (2.6%) |
| Toxin EIA positive (%) | 1/3 (33.3%) | 0/1 (0%) | 1/2 (50.0%) |
| No toxin EIA testing done (%) | 2/3 (66.6%) | 1/1 (100%) | 1/2 (50.0%) |
| GDH– k/n (%) | 37 (45.7%) | 1 (33.3%) | 36 (46.2%) |
| Toxin EIA positive (%) | 2/4 (50.0%) | 1/1 (100%) | 1/3 (33.3%) |
| No toxin EIA testing done (%) | 33/37 (89.2%) | 0/1 (0%) | 33/36 (91.7%) |
| Unknown*– k/n (%) | 16 (19.8%) | 1 (33.3%) | 15 (19.2%) |
| Toxin EIA positive (%) | 4/16 (25.0%) | 1/1 (100%) | 3/15 (20.0%) |
| No toxin EIA testing done (%) | 0/16 (0%) | -- | 0/15 (0%) |
| Clinical testing strategy |  |  |  |
| PCR-Only testing strategy– k/n (%) | 2 (2.5%) | 2 (66.7%) | -- |
| Traditional testing algorithm‡– k/n (%) | 37 (45.7%) | 1 (33.3%) | 36 (46.2%) |
| Toxin EIA positive (%) | 2/4 (50.0%) | 1/1 (100%) | 1/3 (33.3%) |
| Unknown toxin EIA status (%) | 33/37 (89.2%) | -- | 33/36 (91.7%) |
| Reverse testing algorithm‡‡– k/n (%) | 42 (51.9%) | -- | 42 (53.8%) |
| Toxin EIA positive (%) | 17/42 (40.5%) | -- | 17/41 (40.5%) |
| Unknown toxin EIA status (%) | 1/42 (2.4%) | -- | 1/42 (2.4%) |
| Median time from collection to specimen receipt at reference laboratory (IQR) [days] | 187 (168 – 203) | 131 (117 – 145) | 188 (169 – 206) |
|  |  |  |  |
| †: Denominator based on number specimens with toxin testing in each state.  ‡: Traditional testing algorithm: GDH/Toxin EIA arbitrated by PCR  ‡‡: Reverse testing algorithm: PCR followed by Toxin EIA for PCR positives  * Clinical test not fully documented  CBTM: Cary-Blair Transport Medium; PCR: Polymerase Chain Reaction; Dedicated PCR: PCR specifically for *C. difficile*  GDH: Glutamate dehydrogenase; EIA: Enzyme Immunoassay; IQR: Interquartile range; TCCFA: Taurocholate-cycloserine-cefoxitin-fructose agar | | | |

**Supplemental Table 5**

*C. difficile* culture recovery rates of stool specimens from *Clostridioides difficile* test-positive stools identified through Emerging Infections Program surveillance in Colorado and Georgia, January 2020 through December 2022 excluded from the study.

|  | **All available specimens** | **GDH Positive** | **Dedicated PCR** | **Multiplex PCR** | **Unknown Test** |
| --- | --- | --- | --- | --- | --- |
| Total recovery of *C. difficile* | 68/78 (87.2%) | 35/36 (97.2%) | 18/24 (75.0%) | 2/2 (100%) | 13/16 (81.3%) |
| Recovery of *C. difficile* by TCCFA | 60/78(76.9%) | 29/36 (80.6%) | 17/24 (70.8%) | 1/2 (50.0%) | 13/16 (81.3%) |
| Recovery of *C. difficile* by alcohol shock | 8/17 (31.8%) | 6/6 (100%) | 1/7 (14.3%) | 1/1 (100.0%) | 0/3 (0%) |
| Recovery of *C. difficile* in specimens with adequate volume | 68/77 (88.3%) | 35/35 (100.0%) | 18/24 (75.0%) | 2/2 (100%) | 13/16 (81.3%) |

**Supplemental Table 6**

*C. difficile* MLST derived from stool specimens from *Clostridioides difficile* test-positive stools identified through Emerging Infections Program surveillance in Colorado and Georgia, January 2020 through December 2021 excluded from the study.

|  | **All available isolates** | **GDH positive** | **Dedicated PCR** | **Multiplex PCR** | **Unknown clinical test** |
| --- | --- | --- | --- | --- | --- |
| ST1 | 5/40 (12.5%) | 0/22 (0%) | 5/16 (31.3%) | 0/1 (0%) | 0/1 (0.0%) |
| ST2/ST110 | 9/40 (22.5%) | 7/22 (31.8%) | 1/16 (6.3%) | 0/1 (0%) | 1/1 (100%) |
| ST8 | 3/40 (7.5%) | 0/22 (0%) | 2/16 (12.5%) | 1/1 (100%) | 0/1 (0.0%) |
| ST42 | 2/40 (5.0%) | 2/22 (9.1%) | 0/16 (0%) | 0/1 (0%) | 0/1 (0.0%) |
| Other MLST groups* | 21/40 (50.0%) | 13/22 (59.1%) | 8/16 (50.0%) | 0/1 (0%) | 0/1 (0.0%) |
| * Consist of 19 MLST groups | | | | | |

**V.** **Multivariable Logistic Regression Model Variable Selection and Construction**

**Methods**

To assist with variable selection, Cramér's V was assessed between all categorical variables. Variables with a Cramér's V value of ≥0.80 were evaluated to determine which variable should be included in the final model to avoid significant multicollinearity. There was a high degree of association between the clinical diagnostic test and stool transportation medium (i.e., frozen fresh stool or frozen stool in CBTM) because the multiplex PCR test required specimens to be placed into CBTM. Additionally, the clinical diagnostic test and the local laboratory identification (ID) where the clinical testing was done were strongly associated. (Supplemental Figure 2) Due to these findings, the local laboratory ID and the transportation medium (CBTM or fresh stool) were excluded from the multivariable analysis. Additionally, stool specimens that were positive for *C. difficile* by traditional algorithm were excluded from multivariable analysis. These specimens were excluded from analysis to 1) ensure a direct comparison between the *C. difficile* dedicated PCR and the syndromic multiplex PCR, 2) reduce the concern for selection bias as the traditional algorithm only uses a PCR test to arbitrate results for specimens that are already known to be GDH positive and toxin EIA negative. Therefore, any PCR-positive specimen from this group is inherently pre-selected for being toxin EIA-negative, and 3) the traditional algorithm was exclusive to a single study location in Colorado.

**Supplemental Figure 2**

**
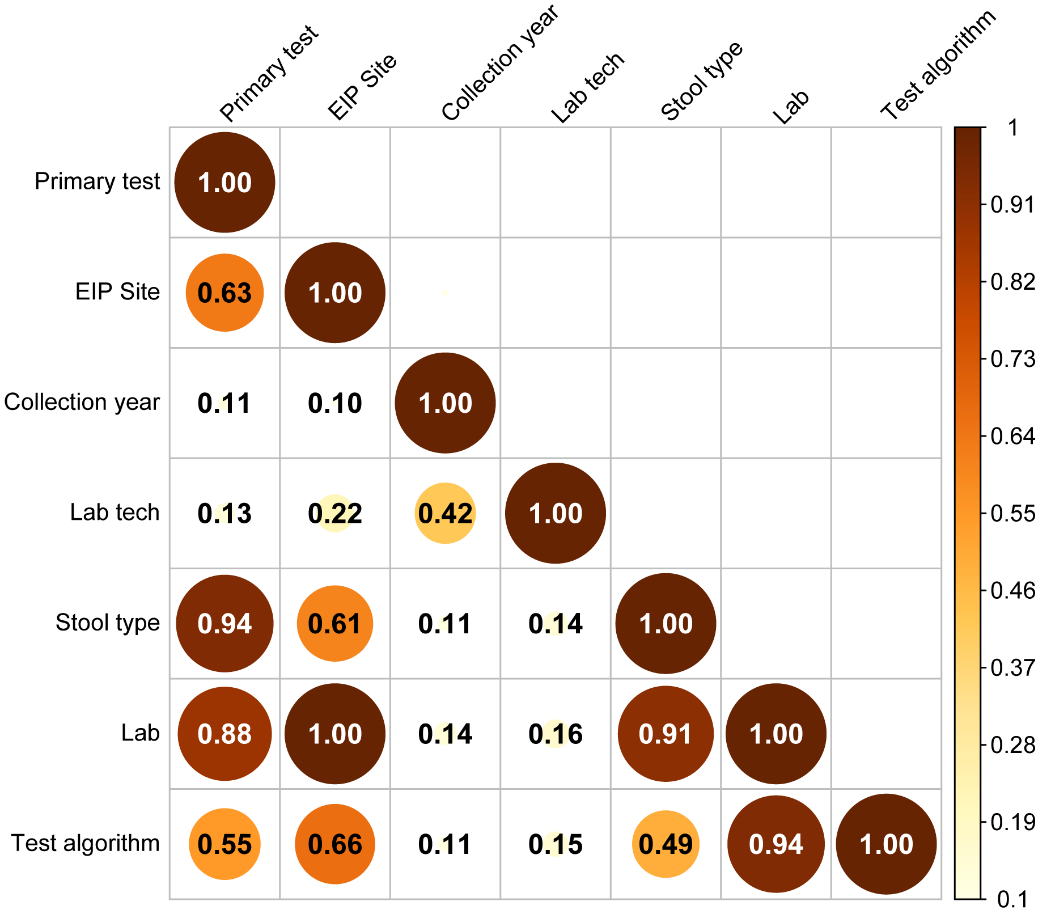
**

**Supplemental Figure 2 Legend.**

Cramér's V values for variables initially selected. Darker color indicates elevated Cramér's V value for the corresponding variables.

The variables included within the first model which included all specimens that were positive for *C. difficile* by a PCR based algorithm (table 3) were the: 1) test used for clinical detection of *C. difficile* (multiplex PCR or dedicated PCR), 2) source from which the stool specimen originated (Colorado or Georgia), 3) test algorithm used for clinical detection (reverse testing algorithm or PCR-only algorithm), 4) year the specimen was collected, and 5) laboratory technician responsible for the culture and recovery of *C. difficile* at the MRL (4 laboratory technicians). This model was re-used for recovery of *C. difficile* by TCCFA plating (Supplemental Table 7) and recovery of *C. difficile* by alcohol shock methods (Supplemental Table 8). Documented diarrhea was excluded from all models as 19.8% (373/1884) did not document if diarrhea was present or not. Goodness-of-fit was confirmed via Hosmer-Lemeshow for all models.

**Logistic regression model for specimens positive by either dedicated PCR or multiplex PCR**

$\text{logit(recovery) = }\beta_{0}\text{ + }\beta_{1}\text{(primary test) + }\beta_{2}\text{(source) + }\beta_{3}\text{(test algorithm) +}\beta_{4}\text{(year) + }\beta_{5}\text{(technician) }$

The variables included within the second model included all specimens that were positive for *C. difficile* by a reverse testing algorithm (table 4) were the: 1) test used for clinical detection of *C. difficile* (multiplex PCR or dedicated PCR, 2) toxin EIA positive status, 3) EIP site from which the stool specimen originated (Colorado or Georgia), 4) year the specimen was collected, and 5) laboratory technician responsible for the culture and recovery of *C. difficile* at the MRL (4 laboratory technicians). Goodness-of-fit was confirmed via Hosmer-Lemeshow.

**Logistic regression model for specimens positive by a reverse testing algorithm**

$\text{logit(recovery) = }\beta_{0}\text{ + }\beta_{1}\text{(primary test) + }\beta_{2}\text{(toxin EIA positive) + }\beta_{3}\text{(source) +}\beta_{4}\text{(year) + }\beta_{5}\text{(technician) }$

The variables included within the third model included all specimens that were positive for *C. difficile* by a PCR-only protocol (table 4) were the: 1) test used for clinical detection of *C. difficile* (multiplex PCR or dedicated PCR, 2) EIP site from which the stool specimen originated (Colorado or Georgia), 3) year the specimen was collected, and 4) laboratory technician responsible for the culture and recovery of *C. difficile* at the MRL (3 primary laboratory technicians and “other laboratory technician” was used when *C. difficile* isolates were recovered by the non-primary technicians during the time of the study [n=2 technicians]). Goodness-of-fit was confirmed via Hosmer-Lemeshow.

**Logistic regression model for specimens positive by a PCR-only protocol**

$\text{logit(recovery) = }\beta_{0}\text{ + }\beta_{1}\text{(primary test) + }\beta_{2}\text{(source) +}\beta_{3}\text{(year) + }\beta_{4}\text{(technician) }$

**Additional logistic regression results for secondary outcomes (TCCFA and alcohol shock recovery)**

Among specimens that were positive by either a multiplex PCR or dedicated PCR (n=1776), the odds of C. difficile recovery was reduced when the clinical detection test was a multiplex PCR (aOR: 0.53; 95% CI: 0.40 – 0.70), and greater among isolates specimens collected from Georgia (aOR: 1.62; 95% CI: 1.62 – 2.24). (Supplemental Table 7)

**Supplemental Table 7**

Multivariable logistic regression model for C. difficile recovery by TCCFA plating for specimens collected by dedicated or multiplex (n=1776)

| Variable | aOR (95% CI) |
| --- | --- |
| Initial clinical diagnostic test |  |
| Dedicated PCR | REF |
| Multiplex PCR | 0.53 (0.40 – 0.70) |
| EIP site |  |
| Colorado | REF |
| Georgia | 1.62 (1.17 – 2.24) |
| Clinical testing algorithm |  |
| PCR-only testing strategy | REF |
| Reverse algorithm testing strategy | 0.93 (0.68 – 1.26) |
| Collection Year |  |
| 2020 | REF |
| 2021 | 1.04 (0.78 – 1.39) |
| 2022 | 0.89 (0.67 – 1.19) |
| MRL laboratory technician |  |
| Laboratory technician #1 | REF |
| Laboratory technician #2 | 1.13 (0.87 – 1.48) |
| Laboratory technician #3 | 0.82 (0.61 – 1.10) |
| Other laboratory technician | 0.99 (0.55 – 1.85) |
| aOR: Adjusted Odds Ratio; CI: Confidence Interval; REF: Reference Group  PCR: Polymerase Chain Reaction; Dedicated PCR: PCR specifically for *C. difficile*;  Multiplex PCR: Syndromic PCR utilized for the detection of multiple gastrointestinal pathogens;  EIA: Enzyme Immunoassay; EIP: Emerging Infections Program  MRL: Microbiological reference Laboratory | |

**Supplemental Table 8**

Multivariable logistic regression model for *C. difficile* recovery by alcohol shock for specimens collected by dedicated or multiplex PCR (n=443)

| Variable | aOR (95% CI) |
| --- | --- |
| Initial clinical diagnostic test |  |
| Dedicated PCR | REF |
| Multiplex PCR | 0.35 (0.19 – 0.62) |
| EIP site |  |
| Colorado | REF |
| Georgia | 0.69 (0.36 – 1.33) |
| Clinical testing algorithm |  |
| PCR-only testing strategy | REF |
| Reverse algorithm testing strategy | 0.73 (0.37 – 1.41) |
| Collection Year |  |
| 2020 | REF |
| 2021 | 0.83 (0.47 – 1.45) |
| 2022 | 1.28 (0.76 – 2.19) |
| MRL laboratory technician |  |
| Laboratory technician #1 | REF |
| Laboratory technician #2 | 0.94 (0.57 – 1.53) |
| Laboratory technician #3 | 1.22 (0.72 – 2.08) |
| Other laboratory technician | 0.83 (0.16 – 3.33) |
| aOR: Adjusted Odds Ratio; CI: Confidence Interval; REF: Reference Group  PCR: Polymerase Chain Reaction; Dedicated PCR: PCR specifically for *C. difficile*;  Multiplex PCR: Syndromic PCR utilized for the detection of multiple gastrointestinal pathogens;  EIA: Enzyme Immunoassay; EIP: Emerging Infections Program  MRL: Microbiological reference Laboratory | |

**VI. Multiplex PCR Coinfection Data**

**Statistical Analysis**

**Logistic regression model for specimens positive by multiplex PCR with multiple positive targets**

1. $\text{logit(recovery) = }\beta_{0}\text{ + }\beta_{1}\text{(coinfection) + }\beta_{2}\text{(source) + }\beta_{4}\text{(year) + }\beta_{5}\text{(technician) }$
2. $\text{logit(recovery) = }\beta_{0}\text{ + }\beta_{1}\text{(bacterial coinfection) + }\beta_{2}\text{(source) + }\beta_{4}\text{(year) + }\beta_{5}\text{(technician) }$
3. $\text{logit(recovery) = }\beta_{0}\text{ + }\beta_{1}\text{(viral coinfection) + }\beta_{2}\text{(source) + }\beta_{4}\text{(year) + }\beta_{5}\text{(technician) }$

Multiplex PCR platforms assess multiple targets which could indicate infection with either bacterial, viral, or protozoal infections. We assessed specimens that were positive for *C. difficile* by multiplex PCR in which the full multiplex PCR results were available (n=780) to assess if co-infection could impact recovery of *C. difficile* by culture. No model produced significant results, and no further analysis was conducted.

There were 183/780 multiplex PCR positive *C. difficile* specimens (23.5%) that were additionally positive for other organisms. Of these 183, 76/183 (41.5%) had a bacterial co-infection, 92/183 (50.3%) had a viral co-infection, 10/183 (5.5%) had a mixed bacterial and viral co-infection, and the remaining 5/183 (2.7%) had a protozoal co-infection.

**VII. MLST data**

**Supplemental Table 9**

Complete MLST for all isolates included in final analysis.

| MLST group | All isolates (n=1024) | Georgia isolates (n=562) | Colorado isolates (n=462) |
| --- | --- | --- | --- |
| 42 | 135 (13.2%) | 62 (11%) | 73 (15.8%) |
| 2 | 104 (10.2%) | 62 (11%) | 42 (9.1%) |
| 8 | 100 (9.8%) | 61 (10.9%) | 39 (8.4%) |
| 1 | 87 (8.5%) | 65 (11.6%) | 22 (4.8%) |
| 110 | 42 (4.1%) | 22 (3.9%) | 20 (4.3%) |
| 34 | 38 (3.7%) | 12 (2.1%) | 26 (5.6%) |
| 3 | 34 (3.3%) | 23 (4.1%) | 11 (2.4%) |
| 43 | 34 (3.3%) | 17 (3%) | 17 (3.7%) |
| 53 | 28 (2.7%) | 17 (3%) | 11 (2.4%) |
| 11 | 26 (2.5%) | 18 (3.2%) | 8 (1.7%) |
| 14 | 25 (2.4%) | 11 (2%) | 14 (3%) |
| 58 | 23 (2.2%) | 12 (2.1%) | 11 (2.4%) |
| 37 | 20 (2%) | 10 (1.8%) | 10 (2.2%) |
| 41 | 20 (2%) | 11 (2%) | 9 (1.9%) |
| 10 | 20 (2%) | 8 (1.4%) | 12 (2.6%) |
| 67 | 20 (2%) | 8 (1.4%) | 12 (2.6%) |
| 55 | 18 (1.8%) | 9 (1.6%) | 9 (1.9%) |
| 28 | 16 (1.6%) | 10 (1.8%) | 6 (1.3%) |
| 6 | 16 (1.6%) | 6 (1.1%) | 10 (2.2%) |
| 4 | 15 (1.5%) | 7 (1.2%) | 8 (1.7%) |
| 15 | 14 (1.4%) | 3 (0.5%) | 11 (2.4%) |
| 54 | 12 (1.2%) | 6 (1.1%) | 6 (1.3%) |
| 26 | 10 (1%) | 2 (0.4%) | 8 (1.7%) |
| 236 | 9 (0.9%) | 6 (1.1%) | 3 (0.6%) |
| 35 | 9 (0.9%) | 5 (0.9%) | 4 (0.9%) |
| 49 | 8 (0.8%) | 7 (1.2%) | 1 (0.2%) |
| 17 | 7 (0.7%) | 5 (0.9%) | 2 (0.4%) |
| 95 | 6 (0.6%) | 2 (0.4%) | 4 (0.9%) |
| Novel profile | 6 (0.6%) | 4 (0.7%) | 2 (0.4%) |
| 21 | 5 (0.5%) | 2 (0.4%) | 3 (0.6%) |
| 16 | 5 (0.5%) | 1 (0.2%) | 4 (0.9%) |
| 44 | 4 (0.4%) | 3 (0.5%) | 1 (0.2%) |
| 188 | 4 (0.4%) | 2 (0.4%) | 2 (0.4%) |
| 190 | 4 (0.4%) | 1 (0.2%) | 3 (0.6%) |
| 103 | 4 (0.4%) | 3 (0.5%) | 1 (0.2%) |
| 47 | 4 (0.4%) | 2 (0.4%) | 2 (0.4%) |
| Novel allele | 4 (0.4%) | 2 (0.4%) | 2 (0.4%) |
| Cryptic clade | 3 (0.3%) | 2 (0.4%) | 1 (0.2%) |
| 45 | 3 (0.3%) | 2 (0.4%) | 1 (0.2%) |
| 36 | 3 (0.3%) | 1 (0.2%) | 2 (0.4%) |
| 9 | 3 (0.3%) | 3 (0.5%) | 0 (0%) |
| 13 | 3 (0.3%) | 1 (0.2%) | 2 (0.4%) |
| 228 | 3 (0.3%) | 2 (0.4%) | 1 (0.2%) |
| 63 | 3 (0.3%) | 2 (0.4%) | 1 (0.2%) |
| 363 | 3 (0.3%) | 0 (0%) | 3 (0.6%) |
| 102 | 3 (0.3%) | 2 (0.4%) | 1 (0.2%) |
| 100 | 3 (0.3%) | 2 (0.4%) | 1 (0.2%) |
| 33 | 2 (0.2%) | 2 (0.4%) | 0 (0%) |
| 12 | 2 (0.2%) | 2 (0.4%) | 0 (0%) |
| 844 | 2 (0.2%) | 2 (0.4%) | 0 (0%) |
| 231 | 2 (0.2%) | 1 (0.2%) | 1 (0.2%) |
| 116 | 2 (0.2%) | 1 (0.2%) | 1 (0.2%) |
| 224 | 2 (0.2%) | 1 (0.2%) | 1 (0.2%) |
| 129 | 2 (0.2%) | 1 (0.2%) | 1 (0.2%) |
| 46 | 2 (0.2%) | 2 (0.4%) | 0 (0%) |
| 1129 | 1 (0.1%) | 0 (0%) | 1 (0.2%) |
| 235 | 1 (0.1%) | 1 (0.2%) | 0 (0%) |
| 395 | 1 (0.1%) | 1 (0.2%) | 0 (0%) |
| 1149 | 1 (0.1%) | 1 (0.2%) | 0 (0%) |
| 1126 | 1 (0.1%) | 1 (0.2%) | 0 (0%) |
| 1137 | 1 (0.1%) | 0 (0%) | 1 (0.2%) |
| 172 | 1 (0.1%) | 1 (0.2%) | 0 (0%) |
| 1134 | 1 (0.1%) | 1 (0.2%) | 0 (0%) |
| 152 | 1 (0.1%) | 1 (0.2%) | 0 (0%) |
| 565 | 1 (0.1%) | 0 (0%) | 1 (0.2%) |
| 1033 | 1 (0.1%) | 1 (0.2%) | 0 (0%) |
| 584 | 1 (0.1%) | 1 (0.2%) | 0 (0%) |
| 222 | 1 (0.1%) | 1 (0.2%) | 0 (0%) |
| 83 | 1 (0.1%) | 1 (0.2%) | 0 (0%) |
| 76 | 1 (0.1%) | 1 (0.2%) | 0 (0%) |
| 642 | 1 (0.1%) | 0 (0%) | 1 (0.2%) |
| 957 | 1 (0.1%) | 0 (0%) | 1 (0.2%) |
| 1147 | 1 (0.1%) | 0 (0%) | 1 (0.2%) |
| 1150 | 1 (0.1%) | 1 (0.2%) | 0 (0%) |
| 19 | 1 (0.1%) | 0 (0%) | 1 (0.2%) |
| 358 | 1 (0.1%) | 0 (0%) | 1 (0.2%) |
| 104 | 1 (0.1%) | 1 (0.2%) | 0 (0%) |
| 232 | 1 (0.1%) | 0 (0%) | 1 (0.2%) |
| 1110 | 1 (0.1%) | 1 (0.2%) | 0 (0%) |
| 51 | 1 (0.1%) | 0 (0%) | 1 (0.2%) |
| 191 | 1 (0.1%) | 0 (0%) | 1 (0.2%) |
| 48 | 1 (0.1%) | 1 (0.2%) | 0 (0%) |
| 139 | 1 (0.1%) | 1 (0.2%) | 0 (0%) |
| 29 | 1 (0.1%) | 1 (0.2%) | 0 (0%) |
| 198 | 1 (0.1%) | 1 (0.2%) | 0 (0%) |
| 176 | 1 (0.1%) | 0 (0%) | 1 (0.2%) |
| 123 | 1 (0.1%) | 0 (0%) | 1 (0.2%) |
| 979 | 1 (0.1%) | 1 (0.2%) | 0 (0%) |
| 868 | 1 (0.1%) | 0 (0%) | 1 (0.2%) |
| 194 | 1 (0.1%) | 1 (0.2%) | 0 (0%) |
| 59 | 1 (0.1%) | 1 (0.2%) | 0 (0%) |
| 606 | 1 (0.1%) | 0 (0%) | 1 (0.2%) |
| 372 | 1 (0.1%) | 1 (0.2%) | 0 (0%) |
| 286 | 1 (0.1%) | 1 (0.2%) | 0 (0%) |
| 325 | 1 (0.1%) | 1 (0.2%) | 0 (0%) |
| 454 | 1 (0.1%) | 1 (0.2%) | 0 (0%) |
| 225 | 1 (0.1%) | 0 (0%) | 1 (0.2%) |

**VIII. Multinomial Logistic Regression Analysis**

**Methods**

Using the same variables as the multivariable logistic regression models, a series multinomial multivariable logistic regression models were constructed to evaluate the relationships between factors which may influence the culture recovery of *C. difficile* with the recovery of ST1, ST2/ST110, ST8, ST 42, and a group labeled as “Other STs” from stool specimens that were wither dedicated PCR-positive or multiplex PCR-positive. Goodness-of-fit was confirmed via Hosmer-Lemeshow.

**Multinomial Logistic Regression Models**

$log(P(\mathrm{ST}_{J}) / P(\mathrm{ST}_{K})) = \beta_{0}J + \beta_{1}J * X_{1} + \beta_{2}J * X_{2} + \ldots+ \beta_{n}J * X_{n}$

**Results**

Multinomial multivariable logistic regression modeling for all specimens that were positive by either a dedicated PCR or multiplex PCR revealed that ST1 was 2.65 times (95% CI: 1.23 - 5.71) more likely to be recovered from specimens that were positive by a reverse testing algorithm when compared to ST42. (Supplemental table 10A) Additionally, ST1 was 2.87 times (95% CI: 1.21 – 6.83) and 2.25 times (95% CI: 1.06 – 4.78) more likely to be recovered from specimens collected from Georgia over Colorado for ST42 and the ‘Other STs’ groups, respectively. (Supplemental Table 10A). ST8 was 2.06 times (95% CI: 1.01 – 4.21) more likely to be recovered from specimens that were positive by a reverse testing algorithm when compared to ST42. (Supplemental table 10C).

When these data were stratified by reverse testing algorithm (n=541), ST1 was 7.17 times (95% CI: 3.40 – 15.11, p <0.01), 2.08 times (95% CI: 1.00-4.36,p=0.051), 2.95 times (95% CI: 1.38 – 6.29; p <0.01), and 3.20 times (95% CI: 1.78 – 5.77; p <0.01) more likely to be toxin EIA positive compared ST2/110, ST8, ST42, and Other STs, respectively. (Supplemental Table 10A) Conversely, ST2 was less likely to be toxin EIA positive when compared to all groups. (Supplemental Table 10B). ST8 was 3.44 times (95% CI: 1.66 – 7.11) more likely to have originated from a toxin EIA positive specimen compared to ST2/110.

**Supplemental Table 10A**

Multinomial multivariate logistic regression model comparing ST1 to ST2/110, ST8, ST42, and Other ST groups

|  | **ST1 vs. ST2/110**† | **ST1 vs. ST8**† | **ST1 vs. ST42**† | **ST1 vs. Other STs**† |
| --- | --- | --- | --- | --- |
|  | aOR (95% CI) | aOR (95% CI) | aOR (95% CI) | aOR (95% CI) |
| Test Algorithm |  |  |  |  |
| PCR-only testing algorithm | REF | REF | REF | REF |
| Reverse testing algorithm | 1.59 (0.74 - 3.40) | 1.28 (0.56 - 2.96) | **2.65 (1.23 - 5.71)*** | 1.67 (0.85 - 3.25) |
| Clinical diagnostic test |  |  |  |  |
| Dedicated PCR | REF | REF | REF | REF |
| Multiplex PCR | 0.9 (0.43 - 1.89) | 0.72 (0.33 - 1.56) | 1.26 (0.58 - 2.76) | 0.84 (0.44 - 1.59) |
| EIP site |  |  |  |  |
| Colorado | REF | REF | REF | REF |
| Georgia | 2.05 (0.87 - 4.81) | 1.75 (0.70 - 4.40) | **2.87 (1.21 - 6.83)*** | **2.25 (1.06 - 4.78)*** |
| Collection Year |  |  |  |  |
| 2020 | REF | REF | REF | REF |
| 2021 | 0.89 (0.51 - 1.56) | 1.15 (0.63 - 2.10) | 0.93 (0.52 - 1.65) | 0.96 (0.60 - 1.56) |
| ST: Sequence type  aOR: Adjusted odds ratio  CI: 95% Confidence interval  †: Reference group  *: p-value <0.05  REF: Reference variable | | | | |

**Supplemental Table 10B**

Multinomial multivariate logistic regression model comparing ST2/110 to ST1, ST8, ST42, and Other ST groups

|  | **ST2/110 vs. ST1**† | **ST2/110 vs. ST8**† | **ST2/110 vs. ST42**† | **ST2/110 vs. Other STs**† |
| --- | --- | --- | --- | --- |
|  | aOR (95% CI) | aOR (95% CI) | aOR (95% CI) | aOR (95% CI) |
| Test Algorithm |  |  |  |  |
| PCR-only testing algorithm | REF | REF | REF | REF |
| Reverse testing algorithm | 0.63 (0.29 - 1.35) | 0.81 (0.40 - 1.64) | 1.67 (0.89 - 3.11) | 1.05 (0.64 - 1.72) |
| Clinical diagnostic test |  |  |  |  |
| Dedicated PCR | REF | REF | REF | REF |
| Multiplex PCR | 1.11 (0.53 - 2.34) | 0.79 (0.40 - 1.57) | 1.40 (0.72 - 2.73) | 0.93 (0.57 - 1.54) |
| EIP site |  |  |  |  |
| Colorado | REF | REF | REF | REF |
| Georgia | 0.49 (0.21 - 1.15) | 0.85 (0.40 - 1.82) | 1.40 (0.71 - 2.78) | 1.10 (0.64 - 1.88) |
| Collection Year |  |  |  |  |
| 2020 | REF | REF | REF | REF |
| 2021 | 1.12 (0.64 - 1.96) | 1.29 (0.76 - 2.18) | 1.04 (0.64 - 1.71) | 1.08 (0.74 - 1.58) |
| ST: Sequence type  aOR: Adjusted odds ratio  CI: 95% Confidence interval  †: Reference group  *: p-value <0.05  REF: Reference variable | | | | |

**Supplemental Table 10C**

Multinomial multivariate logistic regression model comparing ST8 to ST1, ST2/110, ST42, and Other ST groups

|  | **ST8 vs. ST1**† | **ST8 vs. ST2/110**† | **ST8 vs. ST42**† | **ST8 vs. Other STs**† |
| --- | --- | --- | --- | --- |
|  | aOR (95% CI) | aOR (95% CI) | aOR (95% CI) | aOR (95% CI) |
| Test Algorithm |  |  |  |  |
| PCR-only testing algorithm | REF | REF | REF | REF |
| Reverse testing algorithm | 0.78 (0.34 - 1.80) | 1.24 (0.61 - 2.51) | **2.06 (1.01 - 4.21)*** | 1.30 (0.71 - 2.37) |
| Clinical diagnostic test |  |  |  |  |
| Dedicated PCR | REF | REF | REF | REF |
| Multiplex PCR | 1.40 (0.64 - 3.06) | 1.26 (0.64 - 2.48) | 1.76 (0.86 - 3.62) | 1.17 (0.67 - 2.06) |
| EIP site |  |  |  |  |
| Colorado | REF | REF | REF | REF |
| Georgia | 0.57 (0.23 - 1.44) | 1.17 (0.55 - 2.49) | 1.64 (0.76 - 3.54) | 1.29 (0.68 - 2.44) |
| Collection Year |  |  |  |  |
| 2020 | REF | REF | REF | REF |
| 2021 | 0.87 (0.48 - 1.59) | 0.78 (0.46 - 1.31) | 0.81 (0.47 - 1.39) | 0.84 (0.54 - 1.30) |
| ST: Sequence type  aOR: Adjusted odds ratio  CI: 95% Confidence interval  †: Reference group  *: p-value <0.05  REF: Reference variable | | | | |

**Supplemental Table 10D**

Multinomial multivariate logistic regression model comparing ST42 to ST1, ST2/110, ST8, and Other ST groups

|  | **ST42 vs. ST1**† | **ST42 vs. ST2/110**† | **ST42 vs. ST8**† | **ST42 vs. Other STs**† |
| --- | --- | --- | --- | --- |
|  | aOR (95% CI) | aOR (95% CI) | aOR (95% CI) | aOR (95% CI) |
| Test Algorithm |  |  |  |  |
| PCR-only testing algorithm | REF | REF | REF | REF |
| Reverse testing algorithm | **0.38 (0.18 - 0.81)*** | 0.60 (0.32 - 1.12) | **0.49 (0.24 – 0.99)*** | 0.63 (0.38 - 1.04) |
| Clinical diagnostic test |  |  |  |  |
| Dedicated PCR | REF | REF | REF | REF |
| Multiplex PCR | 0.79 (0.36 - 1.74) | 0.71 (0.37 - 1.39) | 0.57 (0.28 - 1.16) | 0.67 (0.39 - 1.15) |
| EIP site |  |  |  |  |
| Colorado | REF | REF | REF | REF |
| Georgia | **0.35 (0.15 - 0.83)*** | 0.71 (0.36 - 1.42) | 0.61 (0.28 - 1.32) | 0.79 (0.45 - 1.36) |
| Collection Year |  |  |  |  |
| 2020 | REF | REF | REF | REF |
| 2021 | 1.07 (0.61 - 1.91) | 0.96 (0.59 - 1.57) | 1.23 (0.72 - 2.12) | 1.04 (0.70 - 1.54) |
| ST: Sequence type  aOR: Adjusted odds ratio  CI: 95% Confidence interval  †: Reference group  *: p-value <0.05  REF: Reference variable | | | | |

**Supplemental Table 11A**

Multinomial multivariate logistic regression model comparing presumed ST1 to ST2/110, ST8, ST42, and Other ST groups stratified by testing algorithm

|  | **Reverse testing algorithm** | | | |  |  | **PCR-only testing algorithm** | | | |
| --- | --- | --- | --- | --- | --- | --- | --- | --- | --- | --- |
|  | **ST1 vs. ST2/110**† | **ST1 vs. ST8**† | **ST1 vs. ST42**† | **ST1 vs. Other STs**† |  |  | **ST1 vs. ST2/110**† | **ST1 vs. ST8**† | **ST1 vs. ST42**† | **ST1 vs. Other STs**† |
|  | aOR  (95% CI) | aOR  (95% CI) | aOR  (95% CI) | aOR  (95% CI) |  |  | aOR  (95% CI) | aOR  (95% CI) | aOR  (95% CI) | aOR  (95% CI) |
| Clinical diagnostic test |  |  |  |  |  | Clinical diagnostic test |  |  |  |  |
| Dedicated PCR | REF | REF | REF | REF |  | Dedicated PCR | REF | REF | REF | REF |
| Multiplex PCR | 1.40  (0.56-3.53) | 1.02  (0.40-2.61) | 1.36  (0.50-3.73) | 1.04  (0.50-2.18) |  | Multiplex PCR | 0.19  (0.03-1.14) | 0.10  (0.01-1.15) | 0.59  (0.13-2.65) | **0.18**  **(0.04-0.78)*** |
| Toxin EIA status |  |  |  |  |  |  |  |  |  |  |
| Toxin EIA negative | REF | REF | REF | REF |  |  |  |  |  |  |
| Toxin EIA positive | **7.17**  **(3.40-15.11)*** | 2.08  (1.00-4.36) | **2.95**  **(1.38-6.29)*** | **3.20**  **(1.78-5.77)*** |  |  |  |  |  |  |
| EIP site |  |  |  |  |  | EIP site |  |  |  |  |
| Colorado | REF | REF | REF | REF |  | Colorado | REF | REF | REF | REF |
| Georgia | 2.36  (0.70-7.99) | 1.99  (0.55-7.720) | 2.45  (0.69-8.70) | **3.17**  **(1.09-9.24)*** |  | Georgia | 0.51  (0.08-3.20) | 0.29  (0.02-3.52) | 1.67  (0.34-8.10) | 0.46  (0.10-2.06) |
| Collection year |  |  |  |  |  | Collection year |  |  |  |  |
| 2020 | REF | REF | REF | REF |  | 2020 | REF | REF | REF | REF |
| 2021 | 0.79  (0.40-1.56) | 1.15  (0.61-2.58) | 0.71  (0.34-1.48) | 0.99  (0.56-1.75) |  | 2021 | 0.85  (0.29-2.50) | 0.90  (0.28-2.87) | 0.99  (0.34-2.82) | 0.80  (0.3-2.12) |
| ST: Sequence type  aOR: Adjusted odds ratio  CI: 95% Confidence interval  †: Reference group  *: p-value <0.05  REF: Reference variable  Reverse testing algorithm: Clinical *C. difficile* testing done by PCR, with positive PCR tested followed by toxin EIA testing  PCR-only testing algorithm: Clinical *C. difficile* testing done by PCR only | | | | | | | | | | |

**Supplemental Table 11B**

Multinomial multivariate logistic regression model comparing presumed ST2/110 to ST1, ST8, ST42, and Other ST groups stratified by testing algorithm

|  | **Reverse toxin EIA testing algorithm** | | | |  |  | **PCR-only testing algorithm** | | | |
| --- | --- | --- | --- | --- | --- | --- | --- | --- | --- | --- |
|  | **ST2/110 vs. ST1**† | **ST2/110 vs. ST8**† | **ST2/110 vs. ST42**† | **ST2/110 vs. Other STs**† |  |  | **ST2/110 vs. ST1**† | **ST2/110 vs. ST8**† | **ST2/110 vs. ST42**† | **ST2/110 vs. Other STs**† |
|  | aOR (95% CI) | aOR (95% CI) | aOR (95% CI) | aOR (95% CI) |  |  | aOR (95% CI) | aOR (95% CI) | aOR (95% CI) | aOR (95% CI) |
| Clinical diagnostic test |  |  |  |  |  | Clinical diagnostic test |  |  |  |  |
| Dedicated PCR | REF | REF | REF | REF |  | Dedicated PCR | REF | REF | REF | REF |
| Multiplex PCR | 0.71  (0.28-1.80) | 0.73  (0.30-1.82) | 0.97  (0.37-2.58) | 0.75  (0.37-1.49) |  | Multiplex PCR | 5.16  (0.88-30.41) | 0.54  (0.05-5.43) | 3.06  (0.79-11.92) | 0.93  (0.25-3.49) |
| Toxin EIA status |  |  |  |  |  |  |  |  |  |  |
| Toxin EIA negative | REF | REF | REF | REF |  |  |  |  |  |  |
| Toxin EIA positive | **0.14**  **(0.07-0.29)*** | **0.79**  **(0.14-0.60)*** | **0.41**  **(0.20-0.86)*** | **0.45**  **(0.25-0.79)*** |  |  |  |  |  |  |
| EIP site |  |  |  |  |  | EIP site |  |  |  |  |
| Colorado | REF | REF | REF | REF |  | Colorado | REF | REF | REF | REF |
| Georgia | 0.42  (0.13-1.43) | 0.84  (0.30-2.34) | 1.04  (0.39-2.78) | 1.34  (0.66-2.74) |  | Georgia | 1.97  (0.31-12.45) | 0.58  (0.05-6.57) | 3.29  (0.74-14.59) | 0.91  (0.22-3.69) |
| Collection year |  |  |  |  |  | Collection year |  |  |  |  |
| 2020 | REF | REF | REF | REF |  | 2020 | REF | REF | REF | REF |
| 2021 | 1.27  (0.64-2.53) | 1.59  (0.80-3.15) | 0.90  (0.45-1.80) | 1.27  (0.77-2.09) |  | 2021 | 1.17  (0.40-3.44) | 1.06  (0.45-2.48) | 1.16  (0.57-2.36) | 0.94  (0.52-1.69) |
| ST: Sequence type  aOR: Adjusted odds ratio  CI: 95% Confidence interval  †: Reference group  *: p-value <0.05  REF: Reference variable  Reverse testing algorithm: Clinical *C. difficile* testing done by PCR, with positive PCR tested followed by toxin EIA testing  PCR-only testing algorithm: Clinical *C. difficile* testing done by PCR only | | | | | | | | | | |

**Supplemental Table 11C**

Multinomial multivariate logistic regression model comparing presumed ST8 to ST1, ST2/110, ST42, and Other ST groups stratified by testing algorithm

|  | **Reverse toxin EIA testing algorithm** | | | |  |  | **PCR-only testing algorithm** | | | |
| --- | --- | --- | --- | --- | --- | --- | --- | --- | --- | --- |
|  | **ST8 vs. ST1**† | **ST8 vs. ST2/110**† | **ST8 vs. ST42**† | **ST8 vs. Other STs**† |  |  | **ST8 vs. ST1**† | **ST8 vs. ST2/110**† | **ST8 vs. ST42**† | **ST8 vs. Other STs**† |
|  | aOR  (95% CI) | aOR  (95% CI) | aOR  (95% CI) | aOR  (95% CI) |  |  | aOR  (95% CI) | aOR  (95% CI) | aOR  (95% CI) | aOR  (95% CI) |
| Clinical diagnostic test |  |  |  |  |  | Clinical diagnostic test |  |  |  |  |
| Dedicated PCR | REF | REF | REF | REF |  | Dedicated PCR | REF | REF | REF | REF |
| Multiplex PCR | 0.97  (0.38-2.48) | 1.36  (0.55-3.39) | 1.33  (0.49-3.61) | 1.02  (0.49-2.10) |  | Multiplex PCR | 9.63  (0.87-106.29) | 1.86  (0.18-18.95) | 5.72  (0.69-47.32) | 1.73  (0.21-14.02) |
| Toxin EIA status |  |  |  |  |  |  |  |  |  |  |
| Toxin EIA negative | REF | REF | REF | REF |  |  |  |  |  |  |
| Toxin EIA positive | 0.48  (0.23-1.00) | **3.44**  **(1.66-7.11)*** | 1.42  (0.68-2.96) | 1.54  (0.87-2.70) |  |  |  |  |  |  |
| EIP site |  |  |  |  |  | EIP site |  |  |  |  |
| Colorado | REF | REF | REF | REF |  | Colorado | REF | REF | REF | REF |
| Georgia | 0.50  (0.14-1.83) | 1.19  (0.43-3.32) | 1.23  (0.41-3.67) | 1.60  (0.68-3.74) |  | Georgia | 3.40  (0.28-40.62) | 1.72  (0.15-19.47) | 5.67  (0.61-52.84) | 1.56  (0.18-13.76) |
| Collection year |  |  |  |  |  | Collection year |  |  |  |  |
| 2020 | REF | REF | REF | REF |  | 2020 | REF | REF | REF | REF |
| 2021 | 0.80  (0.39-1.65) | 0.63  (0.32-1.24) | 0.57  (0.27-1.19) | 0.79  (0.45-1.40) |  | 2021 | 1.11  (0.35-3.55) | 0.95  (0.40-2.23) | 1.10  (0.48-2.52) | 0.89  (0.43-1.84) |
| ST: Sequence type  aOR: Adjusted odds ratio  CI: 95% Confidence interval  †: Reference group  *: p-value <0.05  REF: Reference variable  Reverse testing algorithm: Clinical *C. difficile* testing done by PCR, with positive PCR tested followed by toxin EIA testing  PCR-only testing algorithm: Clinical *C. difficile* testing done by PCR only | | | | | | | | | | |

**Supplemental Table 11D**

Multinomial multivariate logistic regression model comparing presumed ST42 to ST1, ST2/110, ST8, and Other ST groups stratified by testing algorithm

|  | **Reverse toxin EIA testing algorithm** | | | |  |  | **PCR-only testing algorithm** | | | |
| --- | --- | --- | --- | --- | --- | --- | --- | --- | --- | --- |
|  | **ST42 vs. ST1**† | **ST42 vs. ST2/110** | **ST42 vs. ST8**† | **ST42 vs. Other STs**† |  |  | **ST42 vs. ST1**† | **ST42 vs. ST2/110** | **ST42 vs. ST8**† | **ST42 vs. Other STs**† |
|  | aOR (95% CI) | aOR (95% CI) | aOR (95% CI) | aOR (95% CI) |  |  | aOR (95% CI) | aOR (95% CI) | aOR (95% CI) | aOR (95% CI) |
| Clinical diagnostic test |  |  |  |  |  | Clinical diagnostic test |  |  |  |  |
| Dedicated PCR | REF | REF | REF | REF |  | Dedicated PCR | REF | REF | REF | REF |
| Multiplex PCR | 0.73  (0.27-2.01) | 1.03  (0.39-2.72) | 0.75  (0.28-2.05) | 0.77  (0.34-1.72) |  | Multiplex PCR | 1.69  (0.38-7.54) | 0.33  (0.08-1.27) | 0.17  (0.02-1.45) | **0.30**  **(0.12-0.76)*** |
| Toxin EIA status |  |  |  |  |  |  |  |  |  |  |
| Toxin EIA negative | REF | REF | REF | REF |  |  |  |  |  |  |
| Toxin EIA positive | **0.34**  **(0.16-0.72)*** | **2.43**  **(1.16-5.10)*** | 0.71  (0.34-1.48) | 1.09  (0.61-1.95) |  |  |  |  |  |  |
| EIP site |  |  |  |  |  | EIP site |  |  |  |  |
| Colorado | REF | REF | REF | REF |  | Colorado | REF | REF | REF | REF |
| Georgia | 0.41  (0.11-1.45) | 0.96  (0.36-2.59) | 0.81  (0.27-2.42) | 1.30  (0.58-2.92) |  | Georgia | 0.60  (0.12-2.91) | 0.30  (0.07-1.35) | 0.18  (0.02-1.65) | **0.28**  **(0.10-0.78)*** |
| Collection year |  |  |  |  |  | Collection year |  |  |  |  |
| 2020 | REF | REF | REF | REF |  | 2020 | REF | REF | REF | REF |
| 2021 | 1.41  (0.68-2.96) | 1.11  (0.56-2.22) | 1.77  (0.84-3.70) | 1.40  (0.78-2.51) |  | 2021 | 1.01  (0.35-2.90) | 0.86  (0.42-1.76) | 0.91  (0.40-2.10) | 0.81  (0.47-1.41) |
| ST: Sequence type  aOR: Adjusted odds ratio  CI: 95% Confidence interval  †: Reference group  *: p-value <0.05  REF: Reference variable  Reverse testing algorithm: Clinical *C. difficile* testing done by PCR, with positive PCR tested followed by toxin EIA testing  PCR-only testing algorithm: Clinical *C. difficile* testing done by PCR only | | | | | | | | | | |

**IX**. **Supplemental Documentation References**

1. Paulick A, Adamczyk M, Anderson K, et al. Characterization of Clostridioides difficile Isolates Available through the CDC & FDA Antibiotic Resistance Isolate Bank. Microbiol Resour Announc **2021**; 10.

2. Bankevich A, Nurk S, Antipov D, et al. SPAdes: A new genome assembly algorithm and its applications to single-cell sequencing. Journal of Computational Biology **2012**; 19:455–477.

3. Bolger AM, Lohse M, Usadel B. Trimmomatic: a flexible trimmer for Illumina sequence data. Bioinformatics **2014**; 30:2114–2120.

4. Seemann Torsten. MLST: scan contig files against PubMLST typing schemes. 2016. Available at: https://github.com/tseemann/mlst. Accessed 10 April 2024.

5. Jolley KA, Bray JE, Maiden MCJ. Open-access bacterial population genomics: BIGSdb software, the PubMLST.org website and their applications. Wellcome Open Res **2018**; 3:124.

6. Centers for Disease Control and Prevention. Antimicrobial Resistance & Patient Safety Portal - C. difficile Infections. Available at: https://arpsp.cdc.gov/profile/eip/cdi.

7. Kociolek LK, Gerding DN, Hecht DW, Ozer EA. Comparative genomics analysis of Clostridium difficile epidemic strain DH/NAP11/106. Microbes Infect **2018**; 20:245–253.

8. Eyre DW, Davies KA, Davis G, et al. Two Distinct Patterns of Clostridium difficile Diversity Across Europe Indicating Contrasting Routes of Spread. Clinical Infectious Diseases **2018**; 67:1035–1044.

9. Dost I, Abdel-Glil M, Persson S, et al. Genomic study of European Clostridioides difficile ribotype 002/sequence type 8. Microb Genom **2024**; 10.

10. Centers for Disease Control and Prevention. Clostridioides difficile infection (CDI) tracking. Atlanta: Centers for Disease Control and Prevention. 2021. Available at: https://www.cdc.gov/hai/eip/cdiff-tracking.html. Accessed 31 December 2023.
